# Parameters for Irreversible Inactivation of Monoamine Oxidase

**DOI:** 10.3390/molecules25245908

**Published:** 2020-12-13

**Authors:** Rona R. Ramsay, Livia Basile, Antonin Maniquet, Stefanie Hagenow, Matteo Pappalardo, Maria Chiara Saija, Sharon D. Bryant, Alen Albreht, Salvatore Guccione

**Affiliations:** 1 Biomedical Sciences Research Complex, University of St Andrews, St Andrews KY16 8QP, UK; antonin.maniquet@student.unamur.be (A.M.); stefanie.hagenow@hhu.de (S.H.); 2 Department of Health and Drug Sciences, University of Catania, Viale A. Doria 6 ed. 2, I-95125 Catania, Italy; basilelivia@gmail.com (L.B.); mpappala@unict.it (M.P.); mariachiarasaija@hotmail.it (M.C.S.); salvatore.guccione@unict.it (S.G.); 3 Inte:Ligand GmbH, Mariahilferstrasse 74B/11, A-1070 Vienna, Austria; bryant@inteligand.com; 4 Department of Food Chemistry, National Institute of Chemistry, Hajdrihova 19, 1000 Ljubljana, Slovenia; alen.albreht@ki.si

**Keywords:** FAD, irreversible inhibition, enzyme kinetics, computational modeling, pharmacophore, spectrum, adduct

## Abstract

The irreversible inhibitors of monoamine oxidases (MAO) slow neurotransmitter metabolism in depression and neurodegenerative diseases. After oxidation by MAO, hydrazines, cyclopropylamines and propargylamines form a covalent adduct with the flavin cofactor. To assist the design of new compounds to combat neurodegeneration, we have updated the kinetic parameters defining the interaction of these established drugs with human MAO-A and MAO-B and analyzed the required features. The K_i_ values for binding to MAO-A and molecular models show that selectivity is determined by the initial reversible binding. Common to all the irreversible inhibitor classes, the non-covalent 3D-chemical interactions depend on a H-bond donor and hydrophobic-aromatic features within 5.7 angstroms apart and an ionizable amine. Increasing hydrophobic interactions with the aromatic cage through aryl halogenation is important for stabilizing ligands in the binding site for transformation. Good and poor inactivators were investigated using visible spectroscopy and molecular dynamics. The initial binding, close and correctly oriented to the FAD, is important for the oxidation, specifically at the carbon adjacent to the propargyl group. The molecular dynamics study also provides evidence that retention of the allenyl imine product oriented towards FADH^−^ influences the formation of the covalent adduct essential for effective inactivation of MAO.

## 1. Introduction

**Figure 1:**
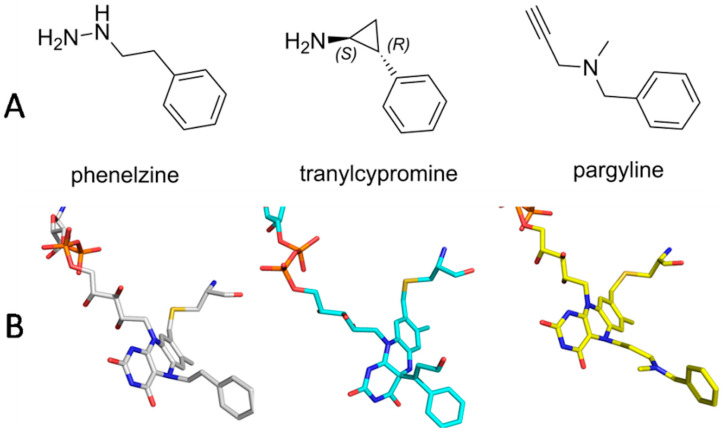
Structures of three non-selective monoamine oxidase (MAO) inhibitor drugs and adducts with flavin adenine dinucleotide (FAD). (**A**) The structures of phenelzine, tranylcypromine and pargyline, representing the three chemical classes of MAO inactivators. (**B**) Adducts of the three compounds covalently bound to FAD derived from X-ray structures of MAO-B. Phenelzine (left, PDB ID: 2VRM) [11] and pargyline (right, PDB ID: 1GOS) [12] both link to N5 of the FAD and tranylcypromine (middle, PDB ID: 2XFU) [13,14] is bonded to C4a of the FAD. Note that tranylcypromine is racemic, the (+)-enantiomer (1*S*,2*R*-trans-2-phenylcyclopropylamine) being 60 times more potent in vitro, although chronic administration of either enantiomer results in MAO-B inhibition in vivo [15].

Monoamine oxidases (MAOs) are modulated clinically by irreversible inhibitors such as phenelzine, tranylcypromine, and selegiline, all of which decrease the metabolism of the major neurotransmitters in the brain. The irreversible inhibitors shown in Figure 1A, except pargyline, have been in use for over 50 years, particularly for depression. Phenelzine and tranylcypromine inhibit both MAO-A and MAO-B and provide patient response rates of better than 50%, as good as or better than tricyclic antidepressants [1], but are used as second-line treatments with dietary restrictions due to side-effects including suicidal ideation, drug–drug and food–drug interactions resulting in seizures and hypotensive crises. Pargyline was discontinued, but selegiline (l-deprenyl), selective for MAO-B inhibition, has proved useful to delay or decrease dosage in l-DOPA treatment of Parkinson’s disease by sparing dopamine [2]. This use has sparked further study into the use of selegiline and other propargylamines not only as MAO-B inhibitors but also in neuroprotection signaling involving the propargyl moiety [3,4,5,6]. The benefit of preserving essential neurotransmitters accompanied by neuroprotection makes the propargyl moiety an attractive fragment to include in multi-target drugs designed to combat neurodegeneration [7,8,9,10]. The ongoing clinical usefulness of irreversible MAO inhibitors prompted this collected report of their inhibitory parameters and common pharmacophore. Then, the interactions of a range of propargylamines and their oxidized products with the active sites of MAO-A and MAO-B were investigated by molecular modeling to define the dynamic interactions that make a good MAO mechanism-based inactivator.

From crystal structures, the active sites of human MAO-A and MAO-B show the same hydrophobic nature but are different in shape. The MAO-B active site entrance is surrounded by hydrophobic amino acid residues (Leu171, Tyr326, Phe168, Ile198, and Ile199), followed by a narrow hydrophobic tunnel leading to the aromatic cage lined by the isoalloxazine of FAD, Tyr398, and Tyr435. The MAO-A active site lacks the constriction seen in MAO-B, allowing bulkier molecules to approach the aromatic cage of FAD, Tyr407, and Tyr444. The crystal structures of MAO-B with the inactivators show that tranylcypromine [13] is attached at C4a of the FAD, but both phenelzine [11] and pargyline [12] form adducts at N5 (Figure 1B). Other propargylamine inactivators similarly form adducts at N5 of the FAD, including clorgyline [16], selegiline [16] and rasagiline [17], and two compounds examined in this work, ASS234 [18] and F2MPA [19]. Formation of the adducts can be measured as the time-dependent onset of inhibition not reversed by dialysis [20] and directly observed by changes in the spectrum of the FAD cofactor [11,18]. The irreversible inhibition by these compounds involves activation by MAO to give an allenyl imine product that reacts chemically to form a covalent bond to the flavin prosthetic group [11,21,22,23]. The mechanisms of adduct formation have been investigated in chemical, structural and computational modeling experiments [11,21,23,24,25,26]. Phenelzine undergoes enzyme-catalyzed conversion to a diazene, which then reacts with oxygen to give an arylalkyl radical that reacts with the flavin to give the N5 adduct [11]. Tranylcypromine modifies the FAD at C4a via a ring-opened radical mechanism [13,14,23,27]. For the propargylamines, the allenyl product of the oxidation reaction reacts with the reduced FADH^−^ [26].

**Scheme 1:**
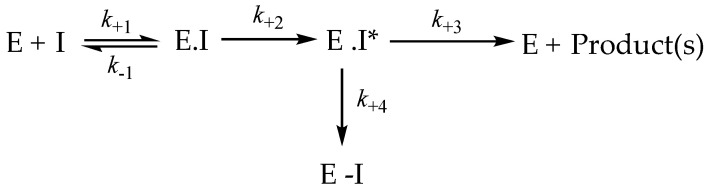
General kinetic scheme for mechanism-based inactivation.

The general kinetic scheme for mechanism-based adduct formation (Scheme 1) shows reversible binding followed by the catalytic step (oxidation, *k*_+2_), the product from which can either dissociate (like a substrate) or form the covalent adduct E-I [20].

**Figure 2:**
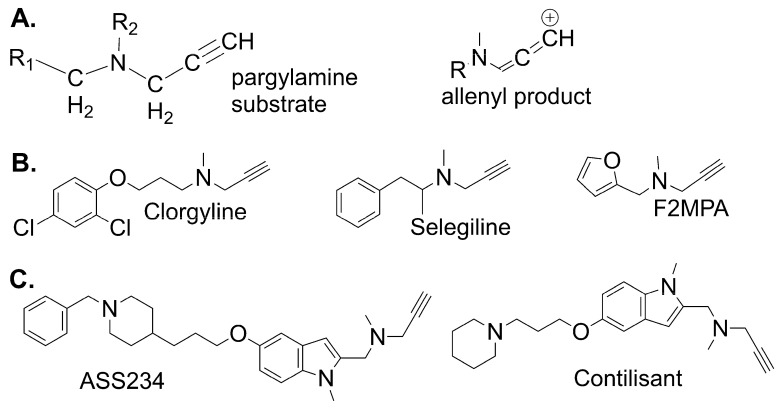
Propargylamine inactivators of MAO. (**A**) General structure of a propargylamine substrate for MAO-A and the allenyl product of oxidation. (**B**) Selective propargylamine inactivators: clorgyline is MAO-A-selective, selegiline and F2MPA are MAO-B-selective. (**C**) New propargylamine compounds incorporating cholinesterase inhibition: ASS234 is MAO-A-selective [18], Contilisant inhibits both MAO-A and MAO-B [36].

The potency of inhibition is determined first by the affinity of the inhibitor for non-covalent (reversible) binding to the enzyme, measured by *K_i_* = *k*_−1_/*k*_+1_. The factors important for maintaining selective, reversible binding have been explored in detail during the design of reversible inhibitors of MAO-A and -B (for example, [28,29]). That the initial oxidation of the amine is slow for all substrates is demonstrated by kinetic isotope effects of between 5 and 12 [30,31,32,33]. Thus, *k*_+2_ determines the rate of formation of the reactive product in the active site. However, if the chemical bond formation (*k*_+4_) is also slow, then the product may dissociate. For example, phenelzine is metabolized as well as slowly inactivating MAO-B [11,30], but clorgyline inactivates MAO-A and selegiline inactivates MAO-B with very little product formation [34]. For these mechanism-based inactivators, the amount of inactivator metabolized divided by the enzyme concentration is the partition ratio, i.e., the number of moles of compound per mole of an enzyme, giving an indication of the efficiency of inactivation. For example, various cyclopropylamine compounds had partition ratios between 3 and 12 [35]. Recent work has shown that the catalytic oxidation and adduct formation steps can be distinguished for one propargylamine compound (ASS234) (Figure 2) using stopped-flow spectrometry, which, with the exploration of the adduct structure and computational modeling, has led to a proposed mechanism for the events in the active site [26].

Early development of the irreversible propargylamine inhibitors focused on empirical measurement and in vivo effects, so the effective conversion to the reactive product with the efficient formation of the covalent attachment has not been studied systematically. Even now, the in vitro screening used in modern high-throughput lead discovery measures only the concentration required for 50% inhibition (IC_50_) at a fixed time point, revealing no information about the rate of inactivation. To aid chemical design and computational screening, an understanding of the determinants of the catalytic and chemical steps is needed. Here we examine the rates of catalytic oxidation and the relative rates of dissociation of product versus adduct formation for the irreversible inhibitors shown in Figure 1 and Figure 2. The kinetics of the spectral changes during adduct formation were used to dissect the enzymic and chemical parts of the reaction. To understand what makes a good inactivator, we incorporated 3D-ligand-based (LB) chemical feature-based pharmacophore models on identifying the 3D-geometries of common chemical features shared by irreversible inhibitors and further analyzed the non-covalent binding partners in MAO binding sites before transformation using structure-based (SB) methods to gain insight into unique interactions features associated with the ligands. In addition, we used molecular dynamics (MD) to probe the ligand proximity and orientation to the flavin before (E.I) and after oxidation (E.I*). Improving mechanism-based inactivators for MAO requires optimization of three factors: an affinity for non-covalent binding to the enzyme, the rate of enzymatic oxidation by MAO (either to the imine form or to the adduct), and the chemical reaction of the reactive product with the enzyme. The MD experiments provide the first insights for their optimization.

## 2. Results and Discussion

### 2.1. Kinetics for Assessment of Mechanism-Based Inhibitors

#### 2.1.1. Reversible Binding of MAO Inactivators

The binding of these mechanism-based inhibitors can be assessed by measuring the inhibition without preincubation, adding the enzyme last to the assay mixture with both substrate and inhibitor. The reversible inhibition of an enzyme by a compound is generally described by the kinetic parameter, K_i_, and it is this parameter that should be used for comparison with binding energies calculated by docking. However, to cope with large numbers of new compounds, the initial assessment is usually by the IC_50_, the inhibitor concentration required to give 50% inhibition. The IC_50_ is not an absolute parameter, but rather is a comparative measure that depends on the type of inhibition and the substrate concentration [37,38]. Table 1 shows the IC_50_ values obtained by adding MAO to the assay containing inhibitor with a substrate at a concentration equal to 2× K_M_ and immediately recording the initial rate. The initial reversible association of an inhibitor with the enzyme is an important factor in the selectivity of the inhibitor, as illustrated by the K_i_ values for MAO-A and -B with clorgyline and selegiline in Table 1. The IC_50_ (without preincubation) of MAO-A for the MAO-A–selective inactivator, clorgyline, is 5357-fold lower than for selegiline, whereas the IC_50_ for clorgyline with MAO-B is 69 times higher than for the MAO-B inactivator, selegiline. Tranylcypromine inhibits MAO-A and MAO-B reversibly at micromolar concentrations and is only 5-fold better for MAO-B when measured as IC_50_ in a coupled assay. Using direct assays, literature K_i_ values for tranylcypromine with MAO-A and MAO-B are approximately equal [39]. The reversible inhibition of MAO by phenelzine is poor (K_i_ is 112 μM for MAO-A, 47 μM for MAO-B), but it does inactivate both MAO-A and -B [11]. MAO-B also oxidizes phenelzine to phenylethylidenehydrazine, and the reduced flavin is reoxidized with concomitant H_2_O_2_ production [11,30]. When MAO-B activity was measured in a direct assay, the K_i_ of 15 μM with phenelzine was 3 times lower than for MAO-A (11).

#### 2.1.2. IC_50_ after Preincubation with MAO

The IC_50_ determined after a fixed incubation time has become a routine way to compare inhibition by new inactivators, although both binding and the rate of inactivator oxidation influence the measurement. Table 2 compares the IC_50_ values after a 30 min preincubation with human MAO expressed in insect cell membranes, then assaying with saturating substrate. The enhancement from the IC_50_ value without preincubation is also given, calculated as the ratio of the values without/with preincubation. These enhancement values differ by compound and enzyme, indicating different rates of reaction after the initial binding to the active site. Notably, the IC_50_ for clorgyline preincubated with MAO-B for 30 min was not different from time zero (enhancement ratio = 1), indicating that very little adduct formation has occurred in the 30 min. However, full irreversible inactivation has been demonstrated in membranes expressing human MAO-B incubated for longer times (see below) and in rat mitochondria (t_1/2_ = 13 min) [34].

#### 2.1.3. Oxidation of Clorgyline Is Required for Adduct Formation

MAO-A was made anaerobic by cycling with vacuum and argon. Figure S1 in Supplementary Information shows the spectral changes that occur when clorgyline is mixed with oxidized or reduced MAO-A in the absence of oxygen (anaerobic). After 30 min, the oxidized MAO-A sample showed a largely increased absorbance at 410 nm (Figure S1) and was fully inactivated. In contrast, no 410 nm increase (adduct formation) was observed in the reduced MAO-A–clorgyline cuvette (Figure S1) and 84% of activity were recovered after 100-fold dilution into an activity assay. After the admission of oxygen to the cuvette, the 420 nm absorbance increased rapidly, accompanied by loss of activity.

#### 2.1.4. Kinetic Constants for Time-Dependent Inactivation

After preincubation with MAO, the inhibition by the compounds in Table 3 was irreversible, and the activity was not restored by dilution 100-fold into an assay mixture containing excess substrate (5× K_M_). The inactivation parameters (k_inact_ and K_I_) for human MAO-A and -B, determined using Kitz-Wilson analysis [41], are shown in Table 3. The rate constant for inactivation by clorgyline is at least 5 times faster than for the non-selective inhibitors, pargyline and tranylcypromine, similar to the four-fold higher rate found for clorgyline on rat MAO-A [34]. The specificity constant (k_inact_/K_I_) for inactivation of purified MAO-A by clorgyline (55 min^−1^·μM^−1^) is greater than for any other MAO-A inactivator. With MAO-B, the specificity constant is highest for selegiline (5.1 min^−1^·μM^−1^), followed by pargyline (0.88 min^−1^·μM^−1^) and tranylcypromine (0.24 min^−1^·μM^−1^). It is apparent that the selectivity of selegiline for MAO-B does not come from the rate constant for inactivation, which is only 2-fold higher than with MAO-A, but from the good reversible binding of selegiline (Table 1) that is 75-fold better for MAO-B.

Recently, developed multi-target designed ligands (MTDL) containing the propargylamine moiety, ASS234 and Contilisant (Figure 2), gave rate constants for inactivation in the same 0.1 to 1 min^−1^ range for both MAO-A and MAO-B, as found for selegiline, pargyline, TCP and phenelzine (Table 3). A small propargylamine, F2MPA, observed to change behavior in rats, inactivated MAO-B but had almost no activity against MAO-A [19].

#### 2.1.5. Partition Ratios

The general kinetic scheme for mechanism-based inactivation (Scheme 1) indicates two possible fates for the product of oxidation by MAO, either release or reaction to form the adduct. The partition ratio was measured by long-term incubation of the enzyme with each compound. Assuming that all the added compound would eventually be oxidized, the added concentration was compared with the moles of enzyme inactivated. For MAO-A, the partition ratios were: clorgyline < 2, pargyline < 5, ASS234 was 7 [18], selegiline 360, and F2MPA > 1000.

### 2.2. D-Chemical Feature-Based-Pharmacophore Interactions

We then looked for the chemical features required for the prediction of good inactivation using both ligand-based and structure-based models.

#### 2.2.1. Ligand-Based Modeling

**Figure 3:**
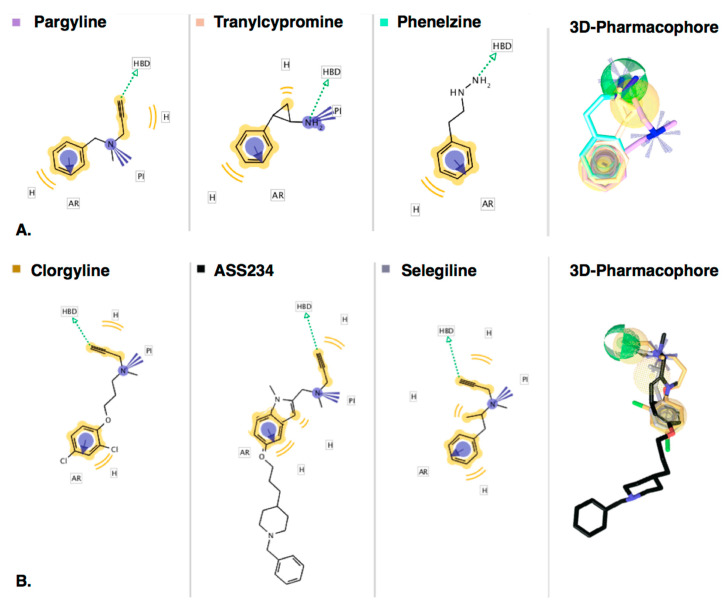
Comparison of chemical features of MAO inactivators derived using ligand-based modeling. 2D-depictions of (**A**) phenelzine (pink), tranylcypromine (beige) and pargyline (cyano) and (**B**) clorgyline (gold), ASS234 (black) and selegiline (gray) aligned to the respective resulting 3D-pharmacophore models (3D-alignments of ligands with the models are to the right). Hydrogens are not displayed. Hydrophobic-aromatic and H-bond donor features were shared by all inactivators in the same 3D-positions. Clorgyline, ASS234, selegiline, and pargyline shared a positive ionizable feature in the same 3D-geometries, whereas the positive ionizable feature of tranylcypromine is in a different geometric space. Hydrophobic (H) features (yellow spheres), aromatic (AR) (blue doughnut-3D or blue circle-2D), positive ionizables (PI) (blue stars) and hydrogen-bond donor (HBD) (green sphere). The pharmacophore models and images were generated using LigandScout 4.4 Expert [43].

Working from the assumption that MAO inactivators must share chemical features in 3D-space if they form adducts with FAD in MAO binding sites, the chemical features that phenelzine, tranylcypromine and pargyline have in common were derived using ligand-based (LB) pharmacophore modeling approaches. A second LB model was derived using the propargylamine inactivators, clorgyline, ASS224 and selegiline. To do this in an unbiased way, multiple 3D-conformations of the ligands were generated without using information from X-ray data for the protein. Alignment experiments of the 3D-geometries based on pharmacophore features (rather than scaffolds) were performed to derive merged 3D-pharmacophore models. The models and 2D-depictions of the corresponding chemical features are shown in Figure 3. The resulting model of the three chemical classes of MAO inactivators (the structures shown in Figure 1) revealed that hydrophobic-aromatic and hydrogen-bond (H-bond) donor features were common to all of them (Figure 3A) with a distance of 5.7 Å apart. While both pargyline and tranylcypromine have positive ionizable features due to the tertiary and primary amines, respectively, those features do not share the same 3D-geometry (Figure 3A). Furthermore, phenelzine does not have a positive ionizable feature due to the chemical nature of the hydrazine. Nevertheless, H-bond donor features associated with the hydrazine terminal NH, the primary amine NH of tranylcypromine, and the terminal alkynyl C-H in pargyline all shared the same geometries (Figure 3A). The alkynyl C-H can donate an H-bond due to a more “acidic” hydrogen atom (higher partial charge) in the presence of attractive electrostatic interactions. Furthermore, clorgyline, selegiline and ASS234 (Figure 3B) have features similar to pargyline, including the hydrophobic-aromatic, positive ionizable and H-bond donor features, all in the same 3D-orientations.

#### 2.2.2. Structure-Based Modeling

To explore the significance of the LB derived chemical features and further identified unique interactions of each ligand that may account for distinct efficacies, the inactivators were evaluated in MAO binding sites derived from X-ray data using structure-based (SB) pharmacophore modeling. Interestingly, two water molecules in close proximity to FAD in several X-ray structures (PDB ID: 2vrm, 2xfu, 4crt, 1s2y, 2byb, 2z5y) displayed H-bond features between O-H of the waters with the isoalloxazine quinone carbonyl oxygens and N5 nitrogen of FAD as well as Gln65, Glu437, Gly56, and Lys298 (Figure S2). The consistency of the observed binding partners in the different MAO sites underscores the importance of the network of hydrogen bonds for structurally stabilizing the geometry between these water molecules and the enzyme for participation in molecular transformations involving ligands, the water themselves and FAD.

**Figure 4:**
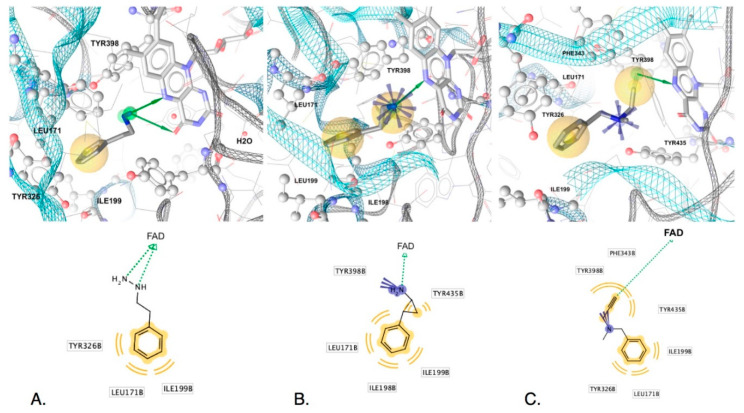
Non-covalent interactions of (**A**) phenelzine, (**B**) tranylcypromine, and (**C**) pargyline in MAO-B (PDB IDs: 2vrm, 2xfu and 1gos). Top images show 3D-interactions in binding sites, and lower ones display 2D-depictions of ligands with features and interaction partners. Hydrogen atoms are not shown. Shown are hydrophobics (yellow spheres), H-bond donors (green arrows), and positive ionizables (blue stars). Interactions were derived using LigandScout 4.4.

**Figure 5:**
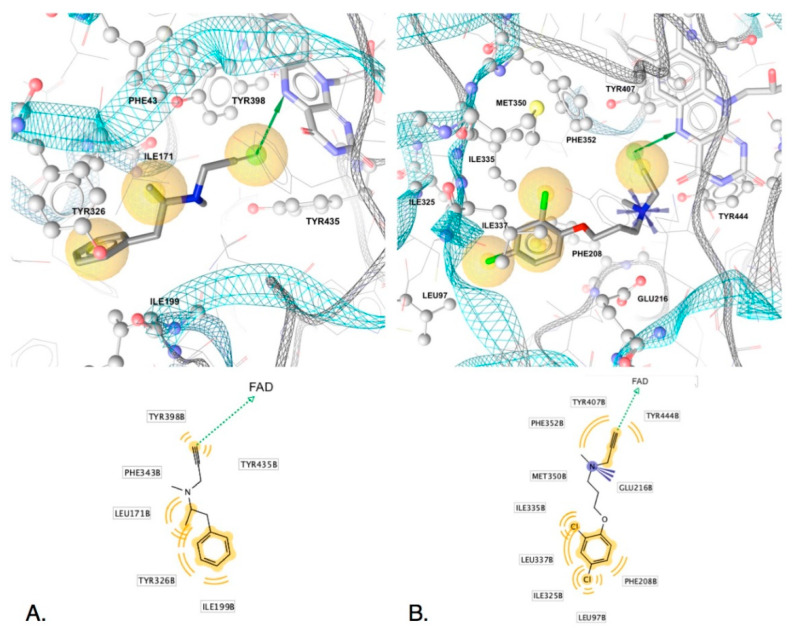
Non-covalent interactions of (**A**) selegiline (L-deprenyl) in MAO-B (PDB ID: 2byb) and (**B**) clorgyline in MAO-A (PDB ID: 2xbr) associated with reversible binding before transformation. Top images show 3D-interactions in binding sites and lower ones display 2D-depictions of ligands with features and interaction partners. Hydrogen atoms are not shown. Hydrophobics (yellow spheres), H-bond donors (green arrows), positive ionizable (blue star). Interactions derived using LigandScout 4.4.

SB-interactions of five of the irreversible inhibitors before transformation derived from X-ray structures (PDB IDs: 2vrm, 2xfu, 1gos, 2byb for MAO-B, and 2bxr for MAO-A) are displayed in Figure 4 and Figure 5. The H-bond donors observed in the LB models (common features) were observed in all of the SB-derived interaction models. Both N-Hs of the nucleophilic hydrazine of phenelzine are in close proximity to FAD and can participate in hydrogen bonding with N5 and C4 carbonyl oxygen (Figure 4A). Furthermore, the N5 of FAD can H-bond with the amine of tranylcypromine (Figure 4B) and the alkynyl terminal C-H of pargyline (Figure 4C), selegiline (Figure 5A) and clorgyline Figure 5B). The terminal C-H of the alkynyl group has a higher positive partial charge resulting in H-bonding with the lone pairs of FAD N5 or alternatively via edge to face aromatic interactions [42]. Similarly, the phenyl groups of each ligand associated with the common hydrophobic-aromatic feature observed in the LB models also resulted in common hydrophobic interaction partners in the SB models, including Leu171, Ile198, Ile199 and Tyr326. In MAO-A, Ile335 and Phe208 are in equivalent positions as Tyr326 and Ile199 in MAO-B, respectively, and also formed hydrophobic interactions with the dichlorinated phenyl group of clorgyline in MAO-A (Figure 5B). Similar interaction partners and feature types observed in the SB models for each of the five ligands indicated similar modes of binding and, furthermore, strengthen the observation that the H-bond donor and hydrophobic-aromatic feature 5.7 angstroms apart represent core features needed for non-covalent binding and chemical transformations in MAO binding sites. In fact, given that phenelzine displays only these interaction features, it represents the minimal interactions required for irreversible inhibition of MAO.

#### 2.2.3. Interactions Conferring Selectivity

Although common features indicate requirements for core activity, unique interaction features may give clues for understanding efficacy, kinetic properties and selectivity of the MAO ligands and serve as suitable vantage points for the design of new inhibitors with improved properties. In the case of clorgyline in MAO-A, the dihalogenated phenyl located in the hydrophobic region of the binding site had a significant impact on the stabilization of the ligand by the aromatic cage since clorgyline engaged the most hydrophobic interaction partners compared to the other inactivators (9 in total compared to 3 in phenelzine, 4 in tranylcypromine, 5 in pargyline and 6 in selegiline). Overall, clorgyline engaged in the most interactions with MAO-A (11 interactions) compared to 5, 6 and 7 total interactions of phenelzine, tranylcypromine, selegiline and pargyline in MAO-B, respectively (Figure 4 and Figure 5). Due to the large number of interactions engaging the aromatic cage, clorgyline is much more stabilized in position ready for transformation compared to the other inhibitors, which may explain its significantly better inactivation of MAO-A compared with tranylcypromine and pargyline and the inactivation with little product dissociation (see below for molecular dynamics support for this).

The positive ionizable features observed in the SB models were associated with unique interaction partners. The amine groups of tranylcypromine and pargyline formed interactions with Tyr398 (Figure 4A and Figure 5B) while clorgyline, in MAO-A, formed an ionic interaction with Glu216 (Figure 5), and no interaction partners were identified for the tertiary amine in selegiline (Figure 5B). However, this does not indicate that there were no potential interaction partners for the tertiary amine in selegiline. Studies involving neuroreceptors aimed towards understanding cationic ligands and the aromatic cage motif indicated that multiple aromatic residues meaningfully contribute to cation interactions, even when there are larger displacements compared to optimal cation-π interaction geometries [43]. This would explain the observed strong inhibition and inactivation parameters of selegiline. In addition, all of the inactivators except phenelzine formed either cation-pi or hydrophobic interactions with Tyr398 (MAO-B) and Tyr 407 (MAO-A) and additional hydrophobic interactions in the same region were observed with Tyr435 and Phe343 (Tyr407 and Phe352 in MAO-A). Tyr398 has been reported to be important for binding, though via aromatic cation-pi interactions or pi-pi interactions—not through H-bonding. Furthermore, Tyr398 (Tyr407-MAO-A) and Tyr435 (Tyr444-MAO-A) have been hypothesized to be involved in orienting substrates in the transformation region of the binding site [44]. The lack of these stabilizing interactions in phenelzine, including the lack of a positive ionizable group, may account for its poor reversible inhibition and slow inactivation of MAO-B compared to the other inhibitors. In addition, the weaker activity of F2MPA may be attributed to the chemical nature of the furan (Figure 2) that has less aromatic character than the phenyl group (present in the other inhibitors), and the furan oxygen is much less basic than the ether oxygen in clorgyline.

### 2.3. Using the Spectrum of MAO-A to Probe Inactivation

#### 2.3.1. Titration of MAO-A with Inactivators

**Figure 6:**
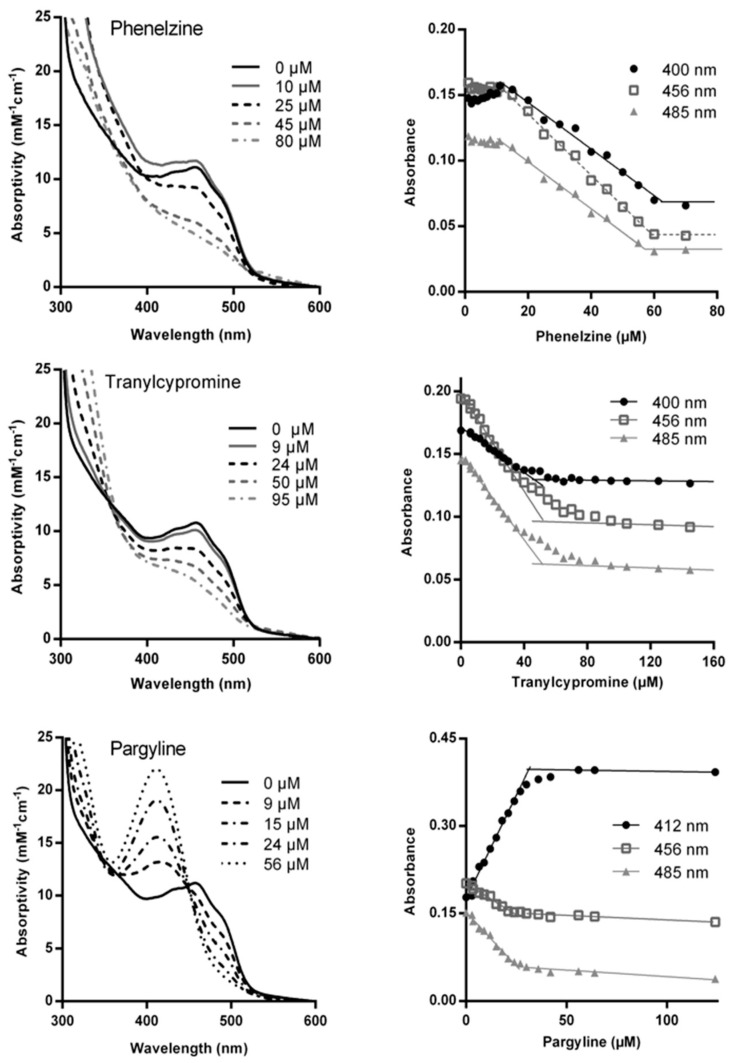
Spectral changes in MAO-A associated with adduct formation. **Left**, selected spectra (plotted as the absorbance of MAO-A at 1 mM in 50 mM potassium phosphate pH 7.5) during the titration; inhibitor (2 μL in water) was added to 400 μL MAO-A and incubated at 20 °C for 15 min before recording each spectrum. **Right**, absorbance changes at selected wavelengths.

Changes in the visible spectrum arising from the FAD have long been used to investigate the redox status and adduct formation. The spectral changes associated with adduct formation in MAO-A were confirmed for the three classical types of mechanism-based inhibitors by adding aliquots of the inhibitor to purified MAO-A and incubating for 15 min at 30 °C before recording the spectrum (Figure 6). As previously observed [11,18,24,44,45,46], the propargylamine adduct is associated with a large increase in the absorbance in the 410 nm region (Figure 6 bottom and Figure S3 in Supplementary Information). No such distinctive feature is seen for tranylcypromine inactivated MAO-A (Figure 6 middle), nor for phenelzine (Figure 6 top). Despite the fact that crystal structures show that all three compounds form adducts with flavin cofactor (Figure 1B), the spectral changes are different. For reference, the extinction coefficients for the changes at key wavelengths, calculated from the spectral titrations shown in Figure 6, are provided in the Supplementary Information Table S1, along with previously published values for reduction of MAO-A in the absence of ligand.

#### 2.3.2. Spectral Changes Distinguish Flavin Reduction from Adduct Formation

**Figure 7:**
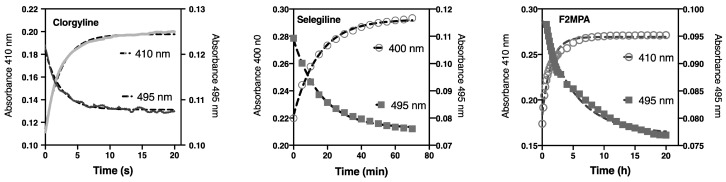
Time courses for absorbance changes in MAO-A incubated anaerobically with propargylamine inactivators. The rate of reduction (decrease in absorbance at 495 nm) and the rate of adduct formation (increase at 410 nm) at 20 °C were measured by stopped-flow spectrometry for clorgyline, but the time courses for selegiline and F2MPA were extracted from a timed collection of spectra in a Unvikon2101 spectrometer. **Left**: clorgyline (50 μM) forms the adduct almost as fast as it reduces MAO-A (19.5 μM). The rates were 29.0 ± 4.1 min^−1^ at 410 nm and 35.9 ± 3.5 min^−1^ at 495 nm. **Middle**: selegiline (500 μM) also forms the adduct and reduces MAO-A (22 µM) at similar monophasic rates (0.016 ± 0.003 and 0.019 ± 0.002 min^−1^, respectively). **Right**: F2MPA (2 mM) with MAO-A (22 μM) gives a monophasic increase at 410 nm but a biphasic decrease in absorbance at 495 nm, requiring more than 21 h for completion.

The observed spectral changes associated with enzyme reduction at 495 nm and with adduct formation at 410 nm were used to follow these two parts of the process of inactivation in a stopped-flow spectrophotometer in order to determine which step was slower (Figure 7). Previously, analysis of spectra recorded every 3 min after the addition of ASS234 to MAO-A revealed a faster initial change at 456 nm than at 410 nm, suggesting that reduction preceded adduct formation [18]. Stopped-flow spectrometry allowed the rates to be measured, confirming that some reduction of MAO-A took place before adduct formation for ASS234 [26]. In contrast, identical rates of change at 495 nm and 410 nm were found in anaerobic experiments for pargyline (0.28 ± 0.02 min^−1^ and 0.26 ± 0.03 min^−1^, respectively, Figure S4) and for clorgyline (29.0 ± 4.1 min^−1^ at 410 nm and 35.9 ± 3.5 min^−1^ at 495 nm, Figure 7) indicating either a concerted reaction of adduct formation or that the rate of MAO-A-catalyzed oxidation (*k*_+2_ in Scheme 1) was slow compared to the adduct formation step (*k*_+4_ in Scheme 1). The rate constant for anaerobic reduction of the FAD in MAO-A by clorgyline was relatively fast at 35.9 min^−1^, whereas reduction by ASS234 was 10 times slower at 3.36 min^−1^. Selegiline was slower yet at 0.019 ± 0.002 min^−1^, and F2MPA was extremely slow at 0.0034 ± 0.0009 min^−1^. The fact that the values for anaerobic reduction (495 nm) of MAO-A by the propargylamines span 4 orders of magnitude indicates that catalytic oxidation of the propargylamines (production of E.I*) is an important discriminating factor.

### 2.4. Exploring Propargylamine Selectivity and Efficacy by Molecular Dynamics

#### 2.4.1. Initial Reversible Binding of Propargylamines to MAO-A

**Figure 8:**
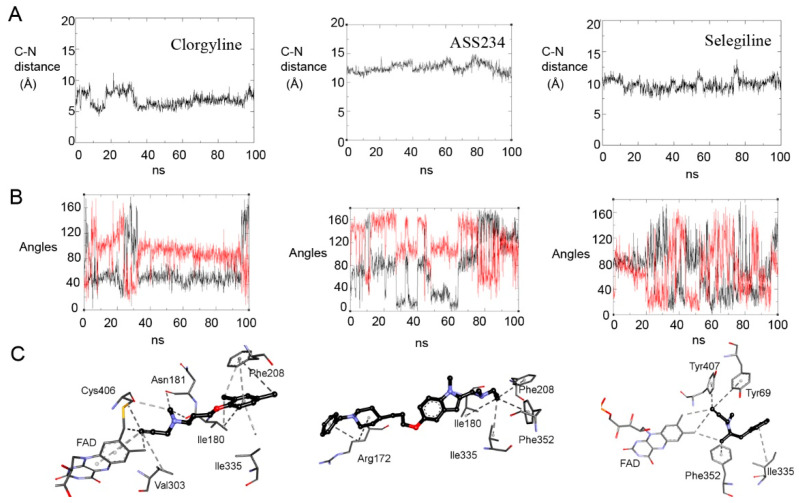
Interactions of propargylamine ligands with oxidized MAO-A. **Left**: clorgyline; **Middle**: ASS234; **Right**: selegiline. (**A**) Poses: clorgyline (at 67 ns of trajectory); ASS234 (at 75 ns); selegiline from its productive pose (at 100 ns). (**B**) Atom plot distances from ligand Cα to N5-FAD calculated over 100 ns of simulation. The selegiline trajectory (right) started from the productive pose in which the propargyl group pointed towards the FAD. (**C**) Angle N5…H-C.

The kinetic data indicate that initial binding is important for selectivity and that there are differences in the reactivity for reduction of MAO-A and for adduct formation. A molecular dynamics (MD) study was used to investigate differences in the various interactions in the active sites of MAO-A and MAO-B that may explain the kinetic behavior. The aim was to define the location of each ligand in the active site and its proximity and orientation relative to the FAD where the oxidation of the ligand takes place. Three propargylamines (clorgyline, selegiline and ASS234) were compared (Figure 8). MD placed the MAO-A-selective clorgyline closest to the N5 of FAD (Figure 8, left). ASS234 was further away (Figure 8, middle). Selegiline with the propargylamine pointing towards the FAD (the productive orientation) shows the Cα offset from N5 (Figure 8, right), but its favored orientation placed the benzyl ring towards the FAD. Details of the interactions shown in Figure 8C are given in Tables S2–S9 and discussed in Supplementary Information.

#### 2.4.2. Distances from N5 to Cα and Orientation for Hydride Transfer 

Analysis of the trajectories was used to define the proximity of Cα of the substrate amine to N5 of the FAD (Figure 8A) and whether the hydrogens on Cα spent time in line between Cα and N5 (Figure 8B), both of which are required for the catalytic hydride transfer [47]. Clorgyline showed the most stable binding at an average distance of 5 Å. The locations of Cα of ASS234 (starting around 12 Å) and of selegiline (around 9.7 Å) varied more, as did the positions of the Cα hydrogens (Figure 8B). The angle subtended from N5.…H-C was stable at around 40° for one hydrogen on Cα in clorgyline and more variable around 100° for the other. The angles varied from 0–160° for ASS234 and selegiline. The mobility is permissive of approach to the calculated 22.4° of the transition state angle in a theoretical model of hydride transfer [47]. The proximity between Cα and the N5 of the FAD revealed by molecular dynamics correlates with the rates of reduction of the flavin in spectral experiments.

#### 2.4.3. Retention of the Product Near the N5 of Reduced MAO-A (FADH^−^)

**Figure 9:**
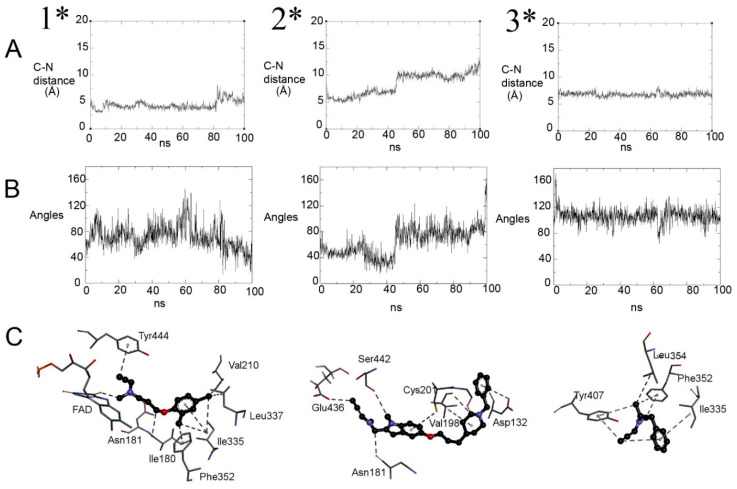
Orientation and movement of allenyl products in the active site of MAO-A. (**A**) Atom plot distances for the imine C-terminal to N5-FADH^−^. (**B**) Angle N5 to C=C=C. (**C**) interactions between allenyl products and reduced MAO-A. Columns from left to right, the compounds are: **left**: oxidized product from clorgyline (1*); **middle**: oxidized product from ASS234 (2*); **right**: oxidized product from selegiline (3*).

After oxidation of a substrate with concomitant reduction of the flavin to FADH^−^, the product would be expected to dissociate. Formation of an adduct, as opposed to dissociation, requires the allenyl form of the product (Figure 1) to remain close to FADH^−^ to allow the nucleophilic attack from the lone pair on N5 of FADH^−^ on the terminal carbon of the allenyl resonance species of the oxidized propargylamine product [26]. The positive charge on this product is likely to exert electrostatic force to prolong residence time [48]. Trajectory analysis of the distance between the terminal carbon of the allenyl product relative to the N5 in FADH^−^ (Figure 9A) shows that the clorgyline product (1*) remains close to the FADH^−^ (4 Å) as expected from its rapid inactivation rate with minimal product formation, and has multiple interactions in the aromatic cage (Figure 9C, left). The imine from ASS234 (2*) had good polar interactions (Figure 9C, middle) with the MAO-A active site holding it close to FADH^−^, but it moved away (5 to 9 Å, Figure 9A) during the 100 ns trajectory, increasing the possibility of dissociation of the product as expected from the partition ratio of 7. Oxidized selegiline (3*) forms interactions further away from the flavin at a distance > 8 Å (Figure 9, right). Integrated with the kinetics, the results imply that retention close to the FADH^−^ is an important factor determining the partition between the release of the product or formation of that covalent adduct.

#### 2.4.4. Selegiline with MAO-B

**Figure 10:**
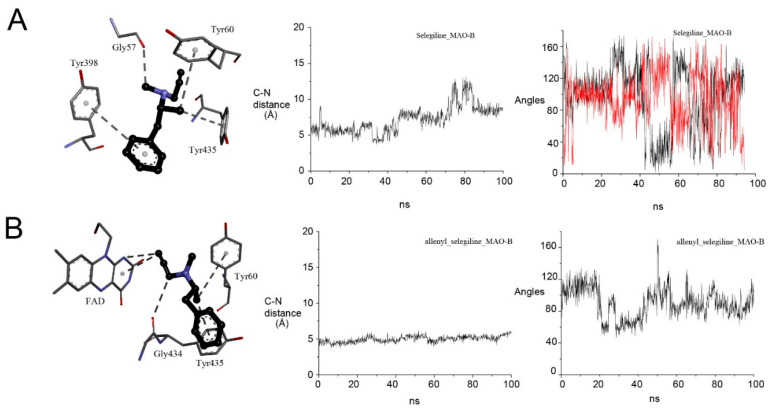
Orientation and movement of selegiline and its allenyl product in the active site of MAO-B. (**A**) Oxidized MAO-B with selegiline; (**B**) reduced MAO-B with the allenyl-imine product from selegiline oxidation. **Left**: interactions in the active site; Middle: atom plot distances of the α-carbon to N5 of FAD (top) or imine C-terminal to N5-FADH^−^ (bottom); **Right**: the angle to FAD or FADH^−^.

The huge difference between MAO-A and MAO-B in binding and inactivation potency for selegiline provides an opportunity to test how the active site interactions result in such a difference. The results of the MD analysis of oxidized MAO-B with selegiline and reduced MAO-B with the allenyl product are shown in Figure 10. Selegiline binds close to the FAD (4–6 Å) for about half of the trajectory, interacting directly with Tyr60, Tyr398 and Tyr438 of the aromatic cage (Figure 10A). The methyl group on the amine forms an H-bond to the main-chain carboxyl of Gly57. This helps orient the CH bond to be oxidized, initially at about 100° to the N5 of the FAD, but it has considerable movement so that either H could be extracted. The allenyl product also has good interactions in the aromatic cage, remaining within 4–5 Å for the whole trajectory (Figure 10B). The terminal carbon chain lies close to perpendicular to the N5 on the *re* side of the FADH^−^.

### 2.5. F2MPA Is Anomalous

#### 2.5.1. F2MPA Interaction with MAO-A

**Figure 11:**
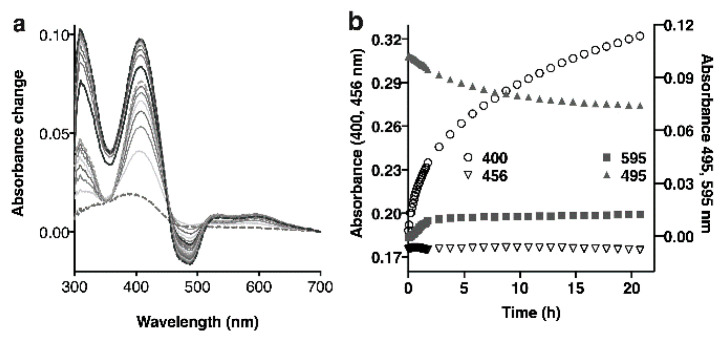
F2MPA partly reduces MAO-A but also forms a charge-transfer complex. Difference spectra were calculated by subtracting the spectrum of anaerobic MAO-A alone from that after incubation with 4. (**a**) Anaerobic F2MPA (2 mM) was added to anaerobic MAO-A (13.4 μM) and spectra collected every 10 min for 2.5 h (spectra at 10 min and 2.5 h shown) and then every hour. (**b**) Time course for reduction of MAO-A (495 nm for bleaching of the FAD; 456 nm is isosbestic), adduct formation (400 nm) and formation of a charge transfer species (595 nm).

**Scheme 2:**
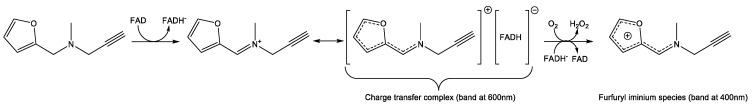
An alternative product from F2MPA oxidation by MAO-A.

The kinetic traces in Figure 7 show that F2MPA is a very poor inactivator of MAO-A, so more work was done to find out why. MAO-A incubated anaerobically for 21.5 h with 0.5 mM F2MPA retained only 7% activity, but overnight aerobic dialysis restored activity to 88%, indicating that minimal covalent adduct had been formed. The slow dissociation suggests tight binding in the complex formed in the absence of oxygen. F2MPA slowly reduced MAO-A under anaerobic conditions, so it is a very poor substrate (Table S2). It also induced an absorbance increase at 600 nm, suggesting a charge transfer complex not seen with any of the other inactivators. The time courses for the relevant wavelengths are shown in Figure 11. In order to find a cause for the 600 nm absorbance, we considered whether oxidation could occur at either carbon adjacent to the amine nitrogen in MAO-A. (MAO-B is inactivated by F2MPA [19], so presumably, the normal allenyl imine product is formed by oxidation at the carbon between the amine and the propargyl group, CαP.) The alternative furfuryl imine product that would result from oxidation at the carbon on the other side of the amine nitrogen (between the amine and the furan ring, CαF) is shown in Scheme 2. Chemical analysis was attempted to detect the putative products of oxidation and molecular modeling to explore the interactions of F2MPA with MAO-A and MAO-B.

#### 2.5.2. Detection of Alternative Products from MAO-A Oxidation of F2MPA

**Scheme 3:**
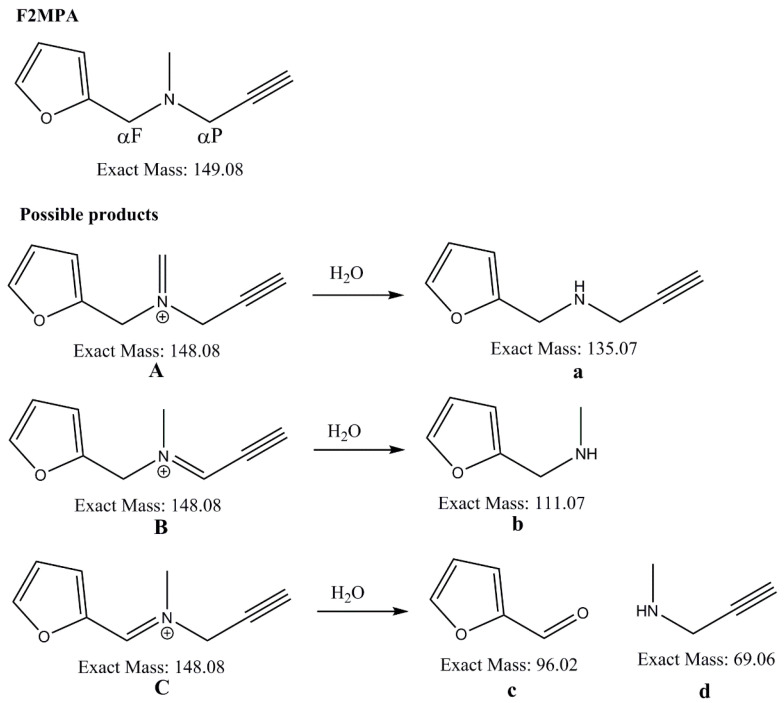
Possible products of F2MPA oxidation.

MAO-A (or MAO B) was incubated with F2MPA for 21 h, and the incubation mixtures were analyzed by HPLC-UV-MS(/MS). Chromatograms were evaluated relative to a control sample, which underwent the same incubation, sample preparation, and analysis, but without the enzyme. The only new species detecting in the samples with MAO (both MAO-A and MAO B) was one with *m*/*z* = 148. Theoretical consideration (Scheme 3) of possible products from MAO catalyzed oxidation of each of the three carbons adjacent to the amine nitrogen gives three species with *m*/*z* = 148, each of which would give different species after hydrolysis. Only the *m*/*z* = 148 species was detected even after extensive alkaline hydrolysis. Thus, it was not possible to determine by analytical chemistry which carbon is oxidized by MAO.

#### 2.5.3. MD Provides Insight into the Differential Selectivity of F2MPA for MAO-A/B and the Spectral Anomalies with MAO-A

**Figure 12:**
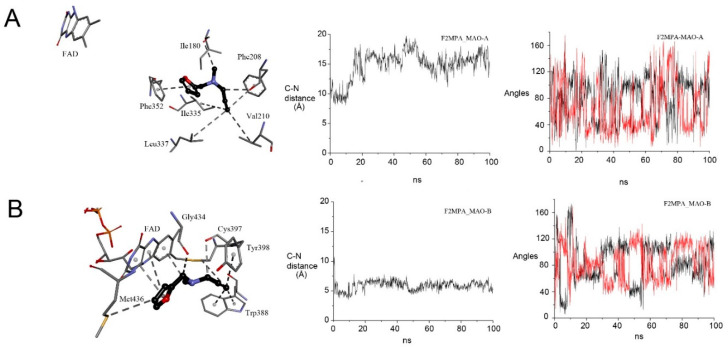
MD analysis of the interaction of F2MPA with MAO-A (**A**) and MAO-B (**B**). **Left**: the location of F2MPA is towards the entrance loop in MAO-A (**A**, **top**), but for MAO-B (**B**, **bottom**) is tight up against the flavin in the aromatic cage. Hydrophobic interactions are shown as gray dotted lines. For MAO-B, the key interactions that contribute to forming the stable complex involve hydrophobic interactions with FAD (by pi-pi T shaped interactions), the residues Trp388, Tyr398 (by pi-alkyl interactions) and Cys397 (by alkyl-alkyl interactions). Ligand is also H-bonded (CH…O) to Gly434, and a pi-sulfur interaction occurs with Met436. **Middle**: CαP-N5 distance. **Right**: the angles C-H-N5 for the hydrogens on Cα at the propargyl side of the amine.

Molecular dynamic simulations were used to explore why F2MPA is so different from the other propargylamines. MD revealed that F2MPA bound well away from the flavin in MAO-A (Figure 12A, left), making catalysis unfavorable as expected from the slow reduction of MAO-A that is observed only under anaerobic conditions. The small F2MPA molecule is mobile within the active site of MAO-A during the trajectory (Figure 12A). The distance between the alpha–carbon (CαP) to N5 of the flavin is never less than 10 Å. It is around 15 Å in the typical pose shown (Figure 12A, left). Likewise, the angle C-H to the N5 in the plane of the flavin ring is highly variable. These results indicate a low probability of oxidation of F2MPA by MAO-A, in agreement with experimental kinetics. In contrast, for MAO-B, F2MPA remains close to the flavin (around 5 Å) bound by multiple hydrophobic interactions and a hydrogen bond (Figure 11B).

**Figure 13:**
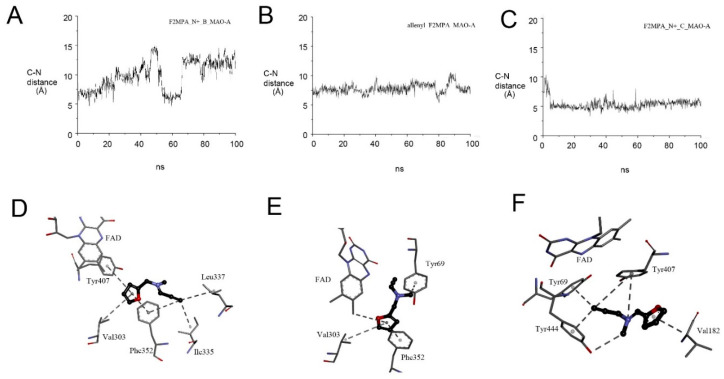
The potential products of F2MPA oxidation bind close to FADH^−^ in reduced MAO-A. Top: the atom plot distances for the imine C-terminal to N5 of FADH^−^. Bottom, the poses in reduced MAO-A. (**A**,**D**) the imine CαP product; (**B**,**E**) the allenyl CαP product; (**C**,**F**) the furfuryl CαF product. Poses taken at (**D**) 90 ns; (**E**) 80 ns; (**F**) 90 ns.

The lack of close proximity of the F2MPA to the FAD in MAO-A makes it impossible to determine from the simulations which of the 2 carbons adjacent to the amine nitrogen is oxidized. The reduction of the flavin in MAO-A but reversibility of the inhibition indicates that oxidation does take place, but not adduct formation. Therefore, the interactions of both putative imine products (B and C in Scheme 3) with reduced MAO-A were investigated by MD (Figure 13). Although we have analyzed the formal imine products with the charge on the nitrogen, it should be noted that imine B (Scheme 3) from oxidation at CαP has three resonance structures, one of which is the allenyl form implicated in adduct formation [26]. The MD results for the imine form B (Figure 13A,D) show it moving away from the flavin, but in contrast, the allenyl resonance form remains in the aromatic cage with the terminal carbon at 6–9 Å from the N_5_H of the FAD (Figure 13B,E). It stacks with the phenyl ring of Phe352 (by pi-pi interaction) and binds Tyr69 by pi-sigma interaction. Other residues involved in the binding of the ligand over the course of the simulation are Phe208, Ile180, Ile335 and Asn181.

The imine C from oxidation at CαF cannot form the allenyl structure but has five resonance forms delocalizing the charge. This furfuryl iminium product **C** (Scheme 3) remains embedded within the aromatic cage (Figure 13C,F), with the furan ring perpendicularly to the isoalloxazine plane of FAD. This structure is stabilized by hydrophobic interactions with the side chain of Tyr407, Tyr444, Tyr69, Val182 (by pi-alkyl interactions). Interestingly, an electrostatic interaction occurs with Tyr407. This close interaction with the furan ring perpendicular to FADH^−^ could be the source of the 600 nm absorbance. In the presence of oxygen, reoxidation to FAD would remove the electrostatic attraction within the charge-transfer complex, allowing the release of the product.

## 3. Conclusions

The design of compounds for mechanism-based inactivation must look at multiple factors. We set out to assemble data to provide insight for the future design of effective mechanism-based inactivators. The kinetics and spectral data are presented to summarize the properties that define the classic mechanism-based inactivators of MAO. Focusing on MAO-A, classic drugs, phenelzine, tranylcypromine and pargyline are all successful due to good binding and fast oxidation. Ligand-based merged feature pharmacophore and SB models indicate clearly that very similar pharmacophore interactions exist for the three main classes of irreversible MAO inhibitors, the hydrazines, the cyclopropylamines and the propargylamines that include a hydrogen bond donor within 5.7 angstroms distance to hydrophobic-aromatic features and the presence of a basic tertiary amine suitable for stabilizing compounds in the aromatic cage of MAO binding site. Furthermore, enhanced hydrophobicity of substitutions in the hydrophobic region, such as halogenated aromatics, will further stabilize compounds designed towards targeting mechanism-based inactivation.

The small propargylamine fragment is of particular interest to ensure MAO inhibition in multi-target compounds [36,49], so we have compared selected propargylamines by kinetics, spectra, and molecular dynamics, showing that orientation and proximity influences both catalysis and adduct formation. The proximity of the terminal carbon of the allenyl product after oxidation of the propargylamine to the reduced FADH^−^ in the MAO-A active site is key to the probability of dissociation versus adduct formation.

**Figure 14:**
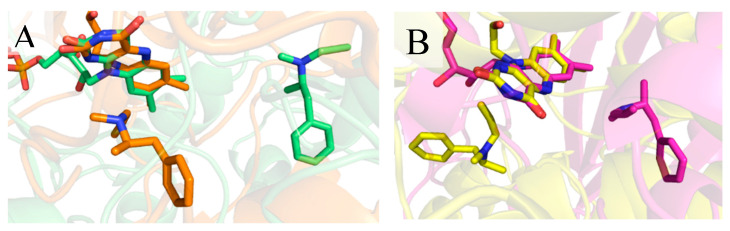
Overlay of selegiline in MAO-A and MAO-B. (**A**) Selegiline locations in the active sites of oxidized MAO, with MAO-A as green sticks and MAO-B as orange sticks. (**B**) Allenyl-selegiline locations in reduced MAO, with MAO-A as magenta sticks and MAO-B as yellow sticks.

Lastly, the selectivity of selegiline for MAO-B, well-established by kinetics, is clearly explained by MD showing the binding, location, and orientation of selegiline close to the FAD in the active site of MAO-B in contrast to its remote and inverted binding in MAO-A (Figure 14). A further improvement of selectivity for use in chronic administration, such as required for Parkinson’s disease therapy, can be had by tuning the binding to decrease the possibility of catalysis in the non-target form. Keeping the required pharmacophore but adjusting the features of the molecule to optimize proximity and orientation in one form of MAO and decrease proximity and force the poor orientation in the other could achieve excellent potency and selectivity.

## 4. Materials and Methods

### 4.1. Kinetic Methods 

#### 4.1.1. Materials

Clorgyline, pargyline, selegiline (l-deprenyl), phenelzine and tranylcypromine were purchased from Sigma-Aldrich, Dorset, UK. Compounds ASS234 [50], Contilisant [36], and F2MPA [19] were synthesized by Dr. J. Marco-Contelles (Madrid, Spain). Commercial human MAO-A and MAO-B expressed in insect cell membranes (Sigma-Aldrich, Dorset, UK) were used for steady-state kinetic experiments. Human MAO-A expressed in yeast cells (*Saccharomyces cerevisiae*) was induced, solubilized and purified as before [51,52].

#### 4.1.2. MAO Assays 

The direct spectrophotometric assay using purified MAO-A (30–50 nM) was added last to a 1 mL final volume of 50 mM potassium phosphate pH 7.5 containing inhibitor (0–100 μM) and 0.3 mM kynuramine (equivalent to 2× K_M_). The increase in absorbance was followed at 314 nm in a Shimadzu UV-2101 spectrophotometer. The coupled assay [53] in black 96-well plates (Eppendorf UK Ltd., Stevenage, UK) used tyramine, Ampliflu™ Red (AR), horseradish peroxidase (HRP) and membrane-bound human MAO-A and MAO-B purchased from Sigma-Aldrich (Dorset, UK). Final concentrations were 50 μM AR, 1 U/mL HRP, and 1 nM enzyme. The substrate was tyramine either at 2× K_M_ (0.8 mM for MAO-A or 0.32 mM for MAO-B) for reversible binding or at 1 mM to assess remaining active MAO after incubation with the inactivators.

#### 4.1.3. Reversible Inhibition

The IC_50_ for reversible inhibition due to the initial binding of each inhibitor was determined from initial rates (without preincubation, i.e., enzyme added last) with 2× K_M_ tyramine (assay concentrations of 0.8 mM for MAO-A and 0.32 mM for MAO-B) fit a three-parameter curve in GraphPad PRISM (v4) [18]. K_i_ values were calculated using the global Michaelis–Menten analysis in PRISM from initial rate data for 6 substrate concentrations (tyramine) at 5 inhibitor concentrations.

#### 4.1.4. Parameters for Irreversible Inhibition

As described previously [18], enzyme and inhibitor were incubated at 20 °C for various times before dilution two-fold by addition of tyramine (to a final concentration of 1 mM) with Ampliflu Red (final concentration 50 μM) and the HRP coupling system to measure the remaining active enzyme by the H_2_O_2_ produced. Rates (as relative fluorescence units per second, rfu/s) were analyzed as the log of the fractional activity remaining compared to control against time to obtain *k_obs_*. The rates (*k_obs_*) plotted against inhibitor concentration gave a rectangular hyperbola that was fitted by nonlinear analysis to give K_I_ and k_inact_ [41].

#### 4.1.5. Partition Ratio

First, the molarity of MAO in the commercial membrane samples was assessed by titration with the appropriate selective inhibitor. MAO-A (membrane-bound, 5 mg.mL^−1^) was diluted 1/500 in 50 mM potassium phosphate pH 7.5 and incubated with 10 concentrations of clorgyline (0–5 μM) at 30 °C for 60 min, then diluted 4-fold with tyramine (to give 1 mM final) and the coupled assay mixture. MAO-B was assessed in the same way, except that selegiline was used and the incubation period was 90 min. The partition ratio was determined by measurement of remaining activity after prolonged incubation with a range of inhibitor concentrations [35,54]. MAO (0.2–0.5 μM) was incubated at 30 °C for up to 10 days with 10 concentrations of the inhibitor. Samples were taken regularly and assayed for the activity remaining after 4-fold dilution into assay medium. When no further inactivation to place in a sample, it is assumed that all the inhibitor has been oxidized. The% remaining enzyme activity was plotted as a function of the ratio of initial inactivator concentration to initial enzyme concentration. The intercept of the linear asymptotes is the partition ratio plus 1. If every molecule oxidized inactivated the enzyme, the partition ratio would be zero.

#### 4.1.6. Visible Spectra of MAO-A with Inactivating Inhibitors

Purified MAO-A was prepared for use by removing dithiothreitol, D-amphetamine, and glycerol used in storage by gel filtration in a G50 spin column equilibrated with 50 mM HEPES pH 7.4. The sample was incubated for 15 min at 30 °C after each addition of a compound to allow completion of the adduct formation before spectra were collected in a Shimazu 2101PC spectrophotometer.

#### 4.1.7. Stopped-Flow Kinetics

Purified human MAO-A was dialyzed against 50 mM HEPES pH 7.5 for 1 h and concentrated to at least 40 μM before loading into the rapid-mixing device syringes. MAO-A (40 μM) was mixed with an equal volume of 50 mM HEPES pH 7.5 containing excess inactivating compound in the stopped-flow spectrophotometer (Applied Photophysics SX20) at 30 °C. For anaerobic experiments, the system was flushed with 10 mM dithionite before rinsing well with buffer containing 30 mM glucose, 1 U/mL glucose oxidase, and 24 U/mL catalase. Both the MAO-A and the compound to be added were flushed with argon before the addition of 30 mM glucose, 1 U/mL glucose oxidase, and 24 U/mL catalase to ensure anaerobiosis during the experiment. Absorbance changes were measured in triplicate at three wavelengths (410, 456, and 495 nm). The absorbance acquisition rate was set at 1000 Hz for the interval 0–0.5 s and 4 Hz for the interval 0.5–1000 s. The absorbance changes were analyzed using single or double exponential fits in the built-in software as appropriate.

### 4.2. Computational Methods

#### 4.2.1. 3D-Pharmacophores

The derivation of chemical feature-based 3D-pharmacophores was done using LigandScout 4.4 Expert (Inte:ligand, Vienna, Austria; http://www.inteligand.com/ligandscout). The structures of the irreversible MAO inhibitors were imported or drawn using the 2D-editor. Low energy 3D-conformations were generated using iCon default Fast settings (maximum of 25 conformations per molecule). 3D-molecular alignments by pharmacophore features were done using the espresso algorithm using the default settings for merged featured pharmacophore creation. For deriving chemical feature interactions from the binding sites, X-ray structures were downloaded from the protein data bank (PDB IDs: 2vrm, 2xfu, 1gos, 2bxr, 2byb, 5mrl, 4crt). Water molecules were optimized using the MMFF94s forcefield as implemented in LigandScout 4.4. The interactions of the water molecules were calculated by moving the waters to the core and FAD and core molecule to the environment and deriving the interactions using the SB algorithm in LigandScout. The waters were moved back to the environment, and only the ligand was returned to the core (binding site) in order to assess interactions between the ligand and its environment (FAD, waters and the surrounding amino acids). The ligands found in the X-ray structures were modified as needed to the original untransformed structures (e.g., for example, phenylethane (PDB: 2vrm) was modified to phenelzine). The final ligand and binding site amino acid side-chains were optimized with the MMFF94s forcefield (default settings in LigandScout 4.4), and chemical feature interactions were calculated using LigandScout’s direct SB approach [55]. In this approach, a chemical feature is added to an SB- pharmacophore model only if there is a complementary binding partner in the binding site. Furthermore, the partner feature must be in the correct 3D-geometries and distances within defined tolerances in order to appear in the model. Default settings in LigandScout 4.4 Expert for feature tolerance definitions were used.

#### 4.2.2. Docking and Molecular Dynamics

The X-ray crystallographic structure of the human MAO-A dimer co-crystallized with 7-methoxy-1-methyl-9h-β-carboline (harmine) (PDB entry: 2Z5X) and MAO-B with 2-(2-benzofuranyl)-2-imidazoline (PDB code: 2XCG) were used for the computational study. First, the co-crystallized inhibitor and the second monomer of MAO-A were deleted using Visual Molecular Dynamics (VMD) v. 1.9.2 [56]. Docking studies were carried out to predict the orientation of the uncharged ligand into the catalytic site using the software AutoDock Vina v. 1.1.2 (11 May 2011) [57] on a PC equipped with an Intel Core i7 processor operating under Linux–Ubuntu 14.04. Docking analysis was performed at the level of the catalytic site of MAO-A and-B with the grid size of 30 × 20 × 20 Å and 20 × 30 × 20 Å, respectively. For each inhibitor, nine poses were generated and ranked according to their scores. The top-scored pose of each inhibitor was chosen as the input for MD simulations after refinement by VMD.

In silico analysis (docking analysis and MD simulation) was carried out for three different MAO-A inhibitors, namely: *N*-[3-(2,4-diclorofenoxy)propyl]-*N*-methyl-prop-2-yn-1-amine (clorgyline, indicated as **1**), *N*-[[5-[3-(1-benzylpiperidin-4-yl)propoxy]-1-methylindol-2-yl] methyl]-*N*-methylprop-2-yn-1-amine (ASS234, indicated as **2**), and (2*R*)-*N*-methyl-1-phenyl-*N*-prop-2-ynylpropan-2-amine (selegiline or l-deprenyl, indicated as **3**) in the neutral and in the oxidized allenyl forms (1*, 2*, 3*). Docking and MD simulation were also carried out for F2MPA (*N*-(furan-2-ylmethyl)-*N*-methylprop-2-yn-1-amine, indicated as **4**) with both MAO-A and MAO-B and two putative oxidized products with MAO-A.

To obtain the allenyl forms, all ligand structures were modified using UCSF Chimera [58] according to the proposed reaction mechanism and changed to the net positively charged allenyl form of the imine by deleting one hydrogen from the C-α. A double bond was added, and the triple bond was changed to a double bond. The partial charge of the terminal C-alkyne was changed to a net positive charge. The FAD structure was modified by adding a hydrogen atom to N5, and the partial charge of N1 was changed to a net negative charge. In accordance with the mechanism proposed by Albreht et al. [26], the nucleophilic attack of FADH^−^ on the allenyl forms of the oxidized ASS234 and clorgyline is thermodynamically driven.

MD simulations were carried out on the NOTUR Linux cluster Stallo (http://hpc.uit.no/en/latest/stallo/stallo.html). The structures were parameterized using the ACPYPE software [59] and confirmed with charmm-gui (http://www.charmm-gui.org/). All systems were solvated in a box of water, and the solutions were ionized with NaCl 0.15 mol/L by using VMD v. 1.9.2 [56]. MD simulations used the NAMD2 software package, version 2.9 [60] with Chemistry at HARvard Macromolecular Mechanics (CHARMM22) as forcefield [61,62]. The TIP3P model for water with a dielectric constant of 1 (ε) was employed [63]. All systems were energy-minimized using the conjugate gradient algorithm [64,65] and heated to 310 °K. The simulations were performed in ensemble number of particles-pressure–temperature kept constant (NPT) at 310 °K and 1 bar. Systems were coupled with a Langevin thermostat and a Langevin barostat to keep temperature and pressure under control. The SHAKE algorithm with a tolerance of 1 × 10^−8^ Å was used to fix the length of the covalent hydrogen bonds [66]. The non-bonded short-range interactions cutoff was set to 12 Å. RMSD values were calculated for all atoms of the protein backbone by using VMD software v. 1.9.2 [56] (https://www.ks.uiuc.edu/Research/vmd/). Analysis of the trajectories of each inhibitor was analyzed over all 100 ns of MD by extracting some frames at the thermal equilibrium, where no big fluctuations were observed using the VMD v. 1.9.2 software and Discovery Studio (DS) Visualizer software (Dassault Systèmes BIOVIA, Discovery Studio Modeling Environment, Release 2017, Dassault Systèmes, San Diego, CA, USA).

## Figures and Tables

**Table 1:** Initial reversible inhibition by mechanism-based inhibitors of MAO.

Inhibitor	MAO-A		MAO-B	
	IC_50_ (μM) AR Assay	K_i_ (μM) Direct	IC_50_ (μM) AR Assay	K_i_ (μM) Direct
Phenelzine	112 ± 28 ^a^ 52.7 ± 5.1	47 ± 2 ^a^	(substrate)	15 ± 2 ^a^ (K_m_ = 215 ± 48)
Tranylcypromine	23.6 ^b^ 54.9 ± 6.2	19 ^c^	4.02 ^b^	16 ^c^
Pargyline	118 ± 21	15 ^d^ (rat)	2.25 ± 0.13	1.8 ^d^ (rat)
Clorgyline	0.042 ± 0.003	0.014 ± 0.001 ^e^ 0.054 ^d^ (rat)	3.65 ± 0.39	58 ^d^ (rat)
Selegiline	225 ± 31	75 ± 11 ^e^ 38 ^d^ (rat)	0.283 ± 0.016	0.97 ^d^ (rat)
ASS234	0.033 ± 0.003	0.053 ± 0.014 ^e^	3.20 ± 0.41	n.d.
Contilisant	1.85 ± 0.21 ^f^	3.3 2.37 ± 0.41	1.94 ± 0.15 ^f^	0.406 ± 0.045

^a^ Binda et al. [11]; ^b^ Malcomson et al. [40]; ^c^ Binda et al. [39]; ^d^ Fowler et al. on rat mitochondria [34]; ^e^ Esteban et al. [18]; ^f^ Bautista-Aguilera et al. [36]; n.d., not determined. All other values are mean ± SD of 2–4 experiments.

**Table 2:** IC_50_ values after 30 min preincubation and the enhancement from the initial IC_50_.

Inhibitor	MAO-A		MAO-B	
	IC_50_ (μM)	Enhancement	IC_50_ (μM)	Enhancement
Phenelzine	4.50 ± 0.72	24.9	Substrate	n/a
Tranylcypromine	0.237 ± 0.061 ^a^	100 ^a^	0.0735 ± 0.0049 ^a^	54.7 ^a^
Pargyline	2.47 ± 0.28	48	0.0077 + 0.0007	292
Clorgyline	0.00042 ± 0.00008	100	3.57 ± 0.36	1.0
Selegiline	0.630 ± 0.06 ^b^	357	0.0040 ± 0.0009	13.3
ASS234	0.00027 ± 0.00003 ^b^	122	0.12 ± 0.02	26.7
Contilisant	0.145 ± 0.010 ^b^	12.8	0.078 ± 0.006 ^b^	24.9

^a^ Malcomson et al. [40]; ^b^ Bautista-Aguilera et al. [36].

**Table 3:** Parameters for inactivation of MAO-A and MAO-B ^a^.

Compound	MAO-A			MAO-B		
	k_inact_ (min^−1^)	K_I_ (μM)	k_inact_/K_I_ (min^−1^μM^−1^)	k_inact_ (min^−1^)	K_I_ (μM)	k_inact_/K_I_ (min^−1^μM^−1^)
Phenelzine	0.12	3.13	0.0383	0.9 ^b^	50 ^b^	0.018
Tranylcypromine	0.776	7.7	0.1008	0.161	0.677	0.238
Pargyline	0.653	22	0.0297	0.35	0.40	0.875
Clorgyline	1.8	0.033	55	0.02 ^c^	1.8 ^c^	0.011
Selegiline	0.252	193	0.0013	0.53	0.103	5.145
ASS234	0.133	0.045	2.956	0.52	1.74	0.299
F2MPA ^c^	0.087	187	0.0005	0.61	135	0.0045
Contilisant	0.39	3	0.1300	0.19	4.6	0.041
MBA364	0.5	4.6	0.1087	0.25	0.055	4.545

^a^ For Scheme 1, *k*_inact_ = *k*_+2_*k*_+4_/(*k*_+2_ + *k*_+3_ + *k*_+4_) and $K_{I}=\left(\frac{k_{-1}+k_{+2}}{k_{+1}}\right)\left(\frac{k_{+3}+k_{+4}}{k_{+2}+k_{+3}+k_{+4}}\right)$; ^b^ From [11]. ^c^ From [19].

## Supplementary Materials

The following are available online, Figure S1: Oxidation of clorgyline is required for adduct formation, Table S1: Molar absorbance changes for reduction or adduct formation with MAO-A, Figure S2: Spectral changes during titration of MAO A with propargylamine inactivators, Figure S3: Stopped-flow traces for the reaction of MAO A with pargyline, Molecular dynamics analysis description, Table S2: Key information from molecular modeling, Tables S3–S9: Interactions between MAO and propargylamine inhibitors –atoms, distances, bonds, and bond types.
