# Supplementary material for: Parameters for Irreversible Inactivation of Monoamine Oxidase
Source: Molecules. 2020 Dec 13;25(24):5908. doi: 10.3390/molecules25245908 (PMC7763263; doi:10.3390/molecules25245908)
Supplement: Supplementary file 1 [file molecules-25-05908-s001.pdf]

Supplementary Information

Parameters for Irreversible Inactivation of Monoamine Oxidase

Rona R. Ramsay [1,*], Livia Basile [2], Antonin Maniquet [1], Stefanie Hagenow [1,†], Matteo Pappalardo [2], Maria Chiara Saija [2,‡], Sharon D. Bryant [3], Alen Albreht [4] and Salvatore Guccione [2]

[1] Biomedical Sciences Research Complex, University of St Andrews, St Andrews KY16 8QP, UK; antonin.maniquet@student.unamur.be (A.M.); stefanie.hagenow@hhu.de (S.H.)
[2] Department of Health and Drug Sciences, University of Catania, Viale A. Doria 6 ed. 2, I-95125 Catania, Italy; basilelivia@gmail.com (L.B.); mpappala@unict.it (M.P.); mariachiarasaija@hotmail.it (M.C.S.); guccione@unict.it (S.G.)
[3] Inte:Ligand GmbH, Mariahilferstrasse 74B/11, A-1070 Vienna, Austria; bryant@inteligand.com
[4] Department of Food Chemistry, National Institute of Chemistry, Hajdrihova 19, 1000 Ljubljana, Slovenia; Alen.Albreht@ki.si
[*] Correspondence: rrr@st-andrews.ac.uk; Tel.: +44-1334-474740
[†] Present Address: Institut fuer Pharmazeutische und Medizinische Chemie, Heinrich-Heine-Universitaet Duesseldorf, Universitaet Strasse. 1, 40225 Duesseldorf, Germany.
[‡] Present Address: Department of Computational Chemistry, J. Heyrovsky Institute of Physical Chemistry, Dolejškova 2155/3, 182 00 Praha 8-Libeň, Czech Republic.

Received: 20 November 2020; Accepted: 9 December 2020; Published: date

Contents

1. Oxidation of clorgyline is required for adduct formation

    Figure S1. Oxidation of clorgyline is required for adduct formation.

2. Water molecule interactions with FAD in MAO-B

    Figure S2. Water molecule interactions in MAO-B X-ray structures

3. Absorbance changes in MAO-A upon reduction or adduct formation

    Table S1. Absorbance changes for reduction or adduct formation with MAO-A.

    Figure S3. Spectral changes during titration of MAO-A with propargylamine inactivators.

    Figure S4. Stopped-flow traces for MAO-A with pargyline.

4. Molecular dynamics analysis

    Table S2. Key information from molecular modeling.

    Tables S3–S9. Interactions between MAO-A and the ligands analyzed.

1. Oxidation of clorgyline is required for adduct formation.

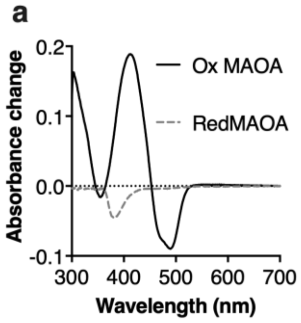

Figure S1. Oxidation of clorgyline is required for adduct formation. MAO-A (9.6 μM) was made anaerobic by cycling between vacuum and argon. (**a**) Difference spectra for the addition of clorgyline (25 μM) to oxidized or reduced anaerobic MAO-A. Clorgyline was added to MAO-A (Ox MAOA, solid line) or to MAO-A pre-reduced by 2-fold excess of the substrate kynuramine (RedMAOA, dashed line) and incubated for 30 min. The Ox MAOA sample was fully inactivated and showed an absorbance increase at 410 nm. No adduct formation was observed in the reduced MAO-A– clorgyline cuvette (RedMAOA), and 84% of activity was recovered after 100-fold dilution into an activity assay.

2. Water molecule interactions with FAD in MAO-B.

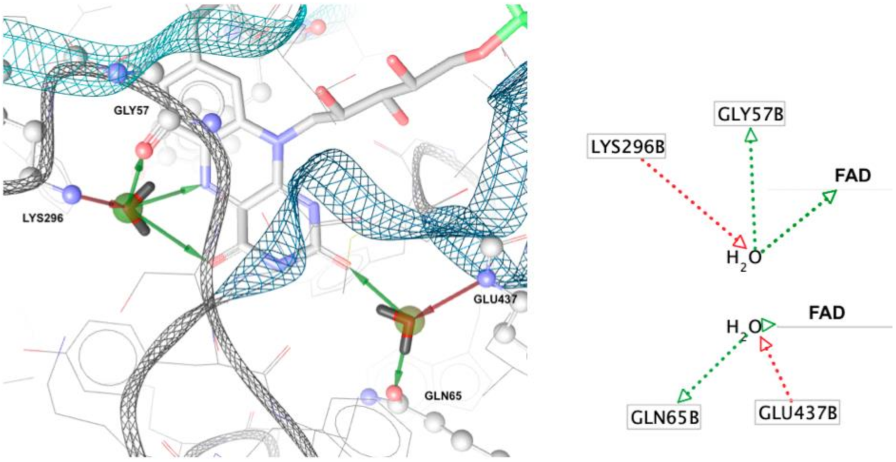

Figure S2. Water molecule interactions with amino acids and FAD in several MAO-B X-ray structures (PDB IDs: 2vrm, 2xfu, 4crt, 1s2y, 2byb, 2z5y). H-bond donors and acceptors represented by green and red arrows, respectively.

3. Absorbance changes in MAO-A upon reduction or adduct formation

Anaerobic incubation with inactivators was associated with a smaller change at 456 nm than for reduction by normal substrates, particularly for the pargyline where the increase in absorbance for the adduct peak at 410 nm counters the decrease expected for reduction. The biggest difference is seen at 410 nm, where the propargylamine adduct increases the absorbance. The absolute value for the maximum absorbance of the final adduct with MAO-A at 410 nm was 25,461 M^{-1} cm^{-1} with pargyline, 23,630 M^{-1} cm^{-1} with clorgyline, and 22,016 M^{-1} cm^{-1} with ASS234. Tranylcypromine and phenelzine adduct formation decreased the absorbance at 410 nm and gave smaller absorbance changes than reduction alone. The change at 485 nm was the least influenced by the adduct structure and was only slightly less than for reduction of MAO-A alone, so this wavelength was used to follow the reduction of MAO-A in the subsequent stopped-flow experiments.

Table S1. Molar absorbance changes for reduction or adduct formation with MAO-A.

	Molar Extinction Coefficient (M^{-1} cm^{-1}) for Difference		
**Compound**	**410 nm**	**456 nm**	**485 nm**
Reduction[a]	−7800	−10,800	−10,700
Reduction [b]	−5600	−10,300	−7800
Reduction by Phenelzine [c]	−6100	−10,500	−8800
**Inactivated MAO-A**			
Phenelzine [c]	−3500	−6300	−4400
Phenelzine	−5737	−8080	−6117
Tranylcypromine	−2870	−6086	−5203
Pargyline	12,946	−3542	−6120
Clorgyline [d]	13,793	−894	−5236
ASS234 [d]	10,853	−1344	−7752

[a] Weyler, W., and Salach, J. I. (1985) Purification and properties of mitochondrial Monoamine-Oxidase type-A from human-placenta, *J. Biol. Chem. 260*, 3199-3207

[b] Hynson, R. M. G., Wouters, J., and Ramsay, R. R. (2003) Monoamine oxidase A inhibitory potency and flavin perturbation are influenced by different aspects of pirlindole inhibitor structure, *Biochemical pharmacology 65*, 1867-1874.

[c] Binda, C., Wang, J., Li, M., Hubalek, F., Mattevi, A., and Edmondson, D. E. (2008) Structural and mechanistic studies of arylalkylhydrazine inhibition of human monoamine oxidases A and B, *Biochemistry 47*, 5616-5625.

[d] Esteban, G., Allan, J., Samadi, A., Mattevi, A., Unzeta, M., Marco-Contelles, J., Binda, C., and Ramsay, R. R. (2014) Kinetic and structural analysis of the irreversible inhibition of human monoamine oxidases by ASS234, a multi-target compound designed for use in Alzheimer's disease, *Biochim Biophys Acta-Proteins and Proteomics 1844*, 1104-1110.

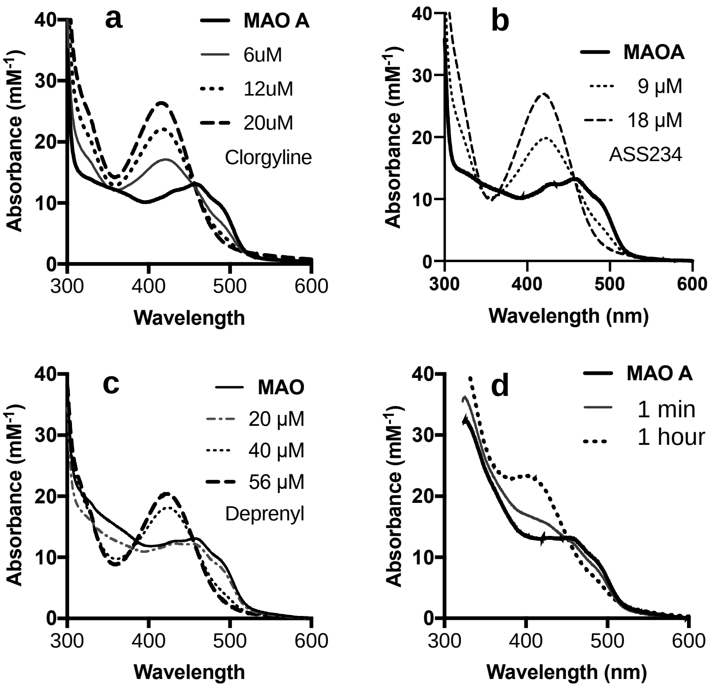

Figure S3. Spectral changes during titration of MAO-A with propargylamine inactivators. Absorbance values were scaled to 1 mM for ease of comparison. Spectra were recorded 10 minutes after each addition when clorgyline and ASS234 gave no further change, but selegiline was still changing. (**a**) 18.9 µM MAO-A with clorgyline; (**b**) 15.6 µM MAO-A with ASS234; (**c**) 13.0 µM MAO-A with selegiline; (**d**) 13.5 µM MAO-A before, then 1 min and 1 hour after addition of 0.5 mM F2MPA.

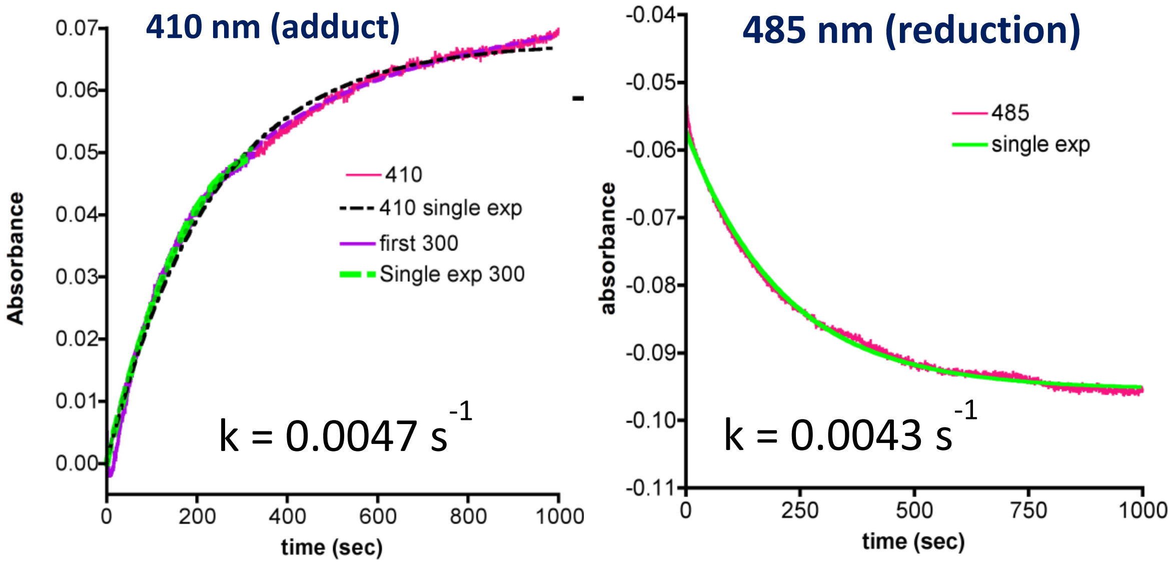

Figure S4. Equal rates of reduction and adduct formation in MAO-A with pargyline. Time courses for absorbance changes in MAO-A mixed anaerobically with propargylamine inactivators. The rate of reduction (decrease in absorbance at 495 nm) and the rate of adduct formation (increase at 410 nm) at 30 °C were measured by stopped-flow spectrometry.

4. Molecular dynamics analysis

Table S2. Key information from molecular modeling

	**Clorgyline**	**ASS234**	**Selegiline** (*with benzyl toward FAD*)[a]	**Selegiline** (*productive complex*)
**Oxidized FAD**				
C_α to N_5 (Å)	4	11.5–13	> 10 with Cα away from FAD	9.7 Å
Angle C_α-H… N_5	100	90–60	Not relevant	140–90
Key contacts	Ile180, Asn181, Phe208, Val303, Ile335, Cys406, isoalloxazine	Arg172, Ile180, Phe208, Ile335, Phe352	Ile160, Phe352, Leu354, Tyr444	Tyr69, Ile335, Phe352, Tyr407 and FAD
**Reduced $FADH^-$**				
Terminal C to N_5 (Å)	4	6–11	11.06	7.5
Angle C=C=C……N_5	0–120 (80)	40–120 (80)	Not relevant	80
Key contacts	Tyr444, Val201, Asn181, Ile180, Phe352, Ile335, Leu337 and FAD	Asp132, Asn181, Val198, Cys201, Glu436, Ser442	Tyr69, Ile180, Phe352, Tyr407	Ile335, Phe352, Leu354 and Tyr407

(a) Selegiline (with benzyl toward FAD) has a benzyl group close to FAD. (b) Selegiline (productive complex) has a propargylamine group close to FAD

4.1. Interactions between MAO-A and ligands.

Based on the kinetic K_i values and the molecular interaction trajectories, we conclude that good binding determines selectivity. The selectivity of reversible binding depends on the interactions listed in the summary Table S2, with details given in Tables S3–S9. For example, hydrogen bonds (CH….O) from different parts of the ligand to the backbone oxygen of Ile180 play a role in keeping both clorgyline (**1**) and ASS234 (**2**) in different positions relative to the FAD. It is the propargylamine

end of ASS234 that bonds to the Ile180 in the located in the middle of the active site, but it is the middle of clorgyline that bonds to Ile180, so that the propargylamine in clorgyline is close to the FAD and forms additional interactions there. Clorgyline gave the most stable interaction in MAO-A active site thanks to multiple interactions with residues close to the flavin and with the isoalloxazine ring itself, holding the Cα carbon within 4 Å of the N5 (as shown in Figure 8 in the main text). ASS234 is held further away from the N5 (> 12 Å), consistent with the 8.5-fold slower rate of reduction than with clorgyline. In contrast to the effective MAO-A inactivators, the best pose for selegiline is inverted, oriented with the benzyl ring allowing π-π stacking with Tyr444, so does not allow oxidation (not shown). For the productive pose of selegiline with the alkyne group inwards (Figure 8C, right), the MD trajectory shows that the Cα averages 10 Å away from the N_5 during the second half of the trajectory (Figure 8A).

Details of the interactions are given in Tables S3–S9. A network of amino acids in the MAO-A active site gives a favorable pattern of interactions with 1. Sidechains of Ile180, Phe208 and Ile335 provide hydrophobic interactions (pi-alkyl interactions) with the benzene ring of the ligand, the main chains of Ile180, Asn181 and Cys406 form hydrogen bonds (CH…O), and side chains of Val303, Cys406, the -CH3 group of the isoalloxazine FAD ring and the isoalloxazine FAD ring give hydrophobic interactions (pi-alkyl and alkyl-alkyl interactions) which stabilize the position of the terminal carbon. In contrast, neither **2** nor **3** interact directly with the FAD. Only one hydrogen bond was found for **2** (CH....O with Ile180 main chain), plus pi-alkyl interactions with Arg172 and hydrophobic interactions with side chains of Phe208, Phe352 (pi-alkyl interactions) and Ile335 (alkyl-alkyl interaction). The interaction pattern of the equilibrated structure of **3** is different from that of **1** and **2**. The side chain of Ile180, Phe352 and Leu354 interact with C8 and C terminal of **3** through hydrophobic interactions (alkyl-alkyl and pi-alkyl interactions), and a pi-pi T shaping interaction occurs between the side chain of Tyr444 and the benzene ring of **3** in its productive pose (Figure 8C right in the main text). For this catalytically important pose, the recurring interactions found between **3** and MAO-A involve the amino acids Tyr69, Ile335, Phe352, Tyr407 and FAD. In detail (Table S4), the side chains of Tyr69, Tyr407, Ile335 and Phe352 interact with the ligand by hydrophobic interactions (pi-alkyl interaction). In addition, C8 of **3** in this productive pose forms hydrophobic interactions with the methyl groups of FAD (alkyl-alkyl interactions).

4.2 Molecular dynamics analysis

4.2.1. Propargylamines with oxidized MAO-A (FAD). The proximity of clorgyline (**1**) to the FAD and the distance between the FAD and ASS234 (**2**) or selegiline (**3**) is evident in the MD trajectories (100 ns) shown in Figure 8 in the main text. Clorgyline maintains a very stable conformation during the whole trajectory (Figure 8A). In the first ≈30 ns the ligand is 12.50 Å from the cofactor FAD, then it moves closer to FAD, and finally, the distance between them decreases to a value of ≈4.5 Å for the remaining runtime of simulation. It is the only ligand lying close to FAD cofactor. For ASS234 (**2**) the distance between the Cα of the propargylamine group and N5 of FAD slightly increases from a value of 11 Å to 13 Å. The distance between selegiline (**3**) and FAD decreases from 16 Å to 10 Å at 50 ns of MD. Unlike **1** and **2**, **3** does not orient its propargylamine group towards the cofactor FAD, but it is positioned in the catalytic site with the benzene ring pointing towards FAD. In the last 30 ns it re-arranges presenting either the propargylamine group or the benzene ring towards FAD, but the Cα-N5 distance calculated at this point is > 10 Å (MD data not shown). However, the propargylamine group pointing towards the flavin is the pose required for the experimentally observed oxidation. The trajectory for **3** from the productive pose is shown in Figure. 8 in the main text. Over 100 ns MD simulation, **3** maintains the productive pose interacting with the FAD. The Cα-N5 distance between the C-terminal of propargylamine group for **3** (productive pose) calculated over 100 ns of simulation shows that **3** maintains an average distance from FAD of 9.7 Å. The MD trajectories clearly rank the probability of oxidation of these compounds by MAO-A (**1 > 2 > 3**) as expected from the kinetic data.

4.2.2. Imines with reduced MAO-A (FADH-). For the imine product from clorgyline (**1*),** the terminal *C-N5* distance fluctuates only slightly over the entire trajectory. Despite this stability, the angle of the terminal carbon chain (C=C=C) to N5 of the flavin is somewhat variable, permissive for adduct formation. Although **2*** is bound to Asp132, Asn181, Val198, Cys201, Glu436 and Ser442 and has no direct interaction with FADH-, the terminal carbon chain angle to N5 is also variable. However, the *C-N* (*C*-terminal alkyne to *N*(5) of FAD) distance of **2*** shows a gradual increase from 11.5 Å to 13 Å over the trajectory, indicating a higher probability of dissociation than for **1*.** Starting with the C=C=C moiety of **3*** close to the FADH- (as it would be immediately after catalytic oxidation from the productive pose), 3* does not change its orientation over the whole 100 ns (Figure 5, bottom, in the main text). The distance between the C-terminal of propargylamine group of 3* and N5 of FADH-, calculated over 100 ns of simulation, maintains an average value of 6.8 Å. The pattern of interactions found for **3*** involves the residues Ile335, Phe352, Leu354 and Tyr407, as listed in Table S9. Sidechains of Phe352, Tyr407 provide for hydrophobic interactions (pi-alkyl interactions) with C8 and C12 of the ligand. In addition, Tyr407 forms a pi-cation interaction with ligand. A H bond occurs between the hydroxy group of Tyr407 (HO…HC) and the ligand. Other hydrophobic interactions were found with Ile335 (pi-alkyl interaction) and Leu354 (alkyl-alkyl interaction). This network of interactions for **3*** keeps the angle to N5 constant at around 80° throughout the trajectory, unlike the variable angles found for **1*** and **2***.

Table S3. MAO-A (FAD) with clorgyline (**1**).

Involved Atoms	Distance Å	Bond	Type
LIG:C6-GLN215:OE1	3.6225	Hydrogen bond	Carbon hydrogen bond
LIG:C9-ILE180:O	3.54412	Hydrogen bond	Carbon hydrogen bond
LIG:C9-ASN181:O	3.3665	Hydrogen bond	Carbon hydrogen bond
LIG:C9-CYS406:O	3.79291	Hydrogen bond	Carbon hydrogen bond
ILE335:CD-LIG	3.46613	Hydrophobic	Pi-Sigma
LIG-PHE208	5.05327	Hydrophobic	Pi-Pi stacked
LIG:Cl-ILE180	3.45552	Hydrophobic	Alkyl
LIG:C12-Val303	5.0132	Hydrophobic	Alkyl
LIG:C12-CYS406	4.14329	Hydrophobic	Alkyl
LIG:C12-FAD1:C53	3.77381	Hydrophobic	Alkyl
LIG:C12-FAD1:C52	3.26364	Hydrophobic	Alkyl
LIG:-ILE180	5.15088	Hydrophobic	Pi-Alkyl
PHE208-LIG:Cl	4.28834	Hydrophobic	Pi-Alkyl
PHE208-LIG:Cl1	5,47197	Hydrophobic	Pi-Alkyl
FAD-LIG:C12	5.41272	Hydrophobic	Pi-Alkyl
FAD-LIG:C12	3.81554	Hydrophobic	Pi-Alkyl

Table S4. MAO-A (FAD) with ASS234 (**2**).

Involved Atoms	Distance Å	Bond	Type
ARG172-LIG	5.36183	Hydrophobic	Alkyl
LIG:C28-ILE180	4.91791	Hydrophobic	Alkyl
LIG:C28-ILE335	4.28961	Hydrophobic	Alkyl
PHE208-LIG:C28	5.1342	Hydrophobic	Pi-Alkyl
PHE352-LIG:C28	4.4287	Hydrophobic	Pi-Alkyl
LIG-ARG172	4.26131	Hydrophobic	Pi-Alkyl
ARG172-LIG	5.36183	Hydrophobic	Alkyl
LIG:C28-ILE180	4.91791	Hydrophobic	Alkyl

Table S5. MAO-A (FAD) with selegiline productive pose (**3**).

Involved Atoms	Distance Å	Bond	Type
LIG:C8C-PHE352	3.47642	Hydrophobic	Pi-Sigma
FAD1:C52-LIG:C8C	3.94425	Hydrophobic	Alkyl
FAD1:C52-LIG:C12	4.04214	Hydrophobic	Alkyl
FAD1:C53-LIG:C12	3.95166	Hydrophobic	Alkyl
TYR69-LIG:C12	5.36782	Hydrophobic	Pi-Alkyl
TYR407-LIG:C12	5.13957	Hydrophobic	Pi-Alkyl
LIG-ILE335	4.74943	Hydrophobic	Pi-Alkyl

Table S6. MAO-A (FAD) with selegiline productive pose (**3**).

Involved Atoms	Distance Å	Bond	Type
LIG:C8C-PHE352	3.47642	Hydrophobic	Pi-Sigma
FAD1:C52-LIG:C8C	3.94425	Hydrophobic	Alkyl
FAD1:C52-LIG:C12	4.04214	Hydrophobic	Alkyl
FAD1:C53-LIG:C12	3.95166	Hydrophobic	Alkyl
TYR69-LIG:C12	5.36782	Hydrophobic	Pi-Alkyl
TYR407-LIG:C12	5.13957	Hydrophobic	Pi-Alkyl
LIG-ILE335	4.74943	Hydrophobic	Pi-Alkyl

Table S7. Reduced MAO-A ($FADH^-$) with clorgyline (**1***).

Involved Atoms	Distance Å	Bond	Type
LIG:C6-ILE180:O	3.52612	Hydrogen bond	Carbon hydrogen bond
LIG:C8-ASN181:OD1	3.38124	Hydrogen bond	Carbon hydrogen bond
LIG:C9-FAD:O17	2.94875	Hydrogen bond	Carbon hydrogen bond
LIG:C12-TYR444	4.25737	Electrostatic	Pi-Cation
LIG:CL-ILE335	4.71317	Hydrophobic	Alkyl
LIG:CL1-VAL210	3.99996	Hydrophobic	Alkyl
LIG:CL1-CYS323	4.70884	Hydrophobic	Alkyl
LIG:CL1-ILE335	4.65263	Hydrophobic	Alkyl
LIG:CL1-LEU337	4.48732	Hydrophobic	Alkyl
PHE352-LIG:CL	3.79187	Hydrophobic	Pi-Alkyl
LIG-ILE335	4.55166	Hydrophobic	Pi-Alkyl

Table S8. Reduced MAO-A ($FADH^-$) with ASS234 product (**2***).

Involved Atoms	Distance Å	Bond	Type
LIG:C03-GLU436:OE1	3.36688	Electrostatic	Attractive Charge
LIG:C6-THR204:OG1	3.61318	Hydrogen bond	Carbon hydrogen bond
LIG:C7-VAL198:O	3.75089	Hydrogen bond	Carbon hydrogen bond
LIG:C7-GLY202:O	3.3769	Hydrogen bond	Carbon hydrogen bond
LIG:C11-THR204:OG1	3.46258	Hydrogen bond	Carbon hydrogen bond
LIG:C23-SER442:O	3.6701	Hydrogen bond	Carbon hydrogen bond
LIG:C24-ASN181:O	3.26343	Hydrogen bond	Carbon hydrogen bond
ASP132:OD1-LIG	4.76211	Electrostatic	Pi-Anion
LIG:C25-TYR197	3.72356	Hydrophobic	Pi-Sigma
CYS201:SG-LIG	5.51147	Other	Pi-Sulfur
O:LIG-A:TYR197	5.64209	Hydrophobic	Pi-Pi stacked
TYR197:C,O;VAL198:N-O:LIG	4.84321	Hydrophobic	Amide-Pi stacked
VAL198:C,O;LYS199:N-O:LIG	4.61287	Hydrophobic	Amide-Pi stacked

A:VAL198-O:LIG	4.71708	Hydrophobic	Alkyl

Table S9. Reduced MAO-A ($FADH^-$) with selegiline productive pose product (**3*).**

**Involved Atoms**	**Distance Å**	**Bond**	**Type**
LIG:C12-TYR407	4.16998	Electrostatic	Pi-Cation
LIG:C12-LIG	4.87152	Electrostatic	Pi-Cation
LIG:C9N-PHE352	3.76146	Hydrophobic	Pi-Sigma
LIG:C8C-LEU354	5.08109	Hydrophobic	Alkyl
PHE352-LIG:C8C	4.94203	Hydrophobic	Pi-Alkyl
TYR407-LIG:C8C	4.97661	Hydrophobic	Pi-Alkyl
LIG-ILE335	4.77563	Hydrophobic	Pi-Alkyl
